# 3D‐Printed Lattice Structures for Sound Absorption: Current Progress, Mechanisms and Models, Structural‐Property Relationships, and Future Outlook

**DOI:** 10.1002/advs.202305232

**Published:** 2023-11-23

**Authors:** Xinwei Li, Jun Wei Chua, Xiang Yu, Zhendong Li, Miao Zhao, Zhonggang Wang, Wei Zhai

**Affiliations:** ^1^ Faculty of Science, Agriculture, and Engineering Newcastle University Singapore 567739 Singapore; ^2^ Department of Mechanical Engineering National University of Singapore Singapore 117575 Singapore; ^3^ Department of Mechanical Engineering The Hong Kong Polytechnic University Hong Kong Hong Kong SAR 999077 China; ^4^ School of Traffic & Transportation Engineering Central South University Changsha 410017 P. R. China

**Keywords:** acoustics modelling, lattice structure, sound absorption, structural‐property relationship, 3D printing

## Abstract

The reduction of noises, achieved through absorption, is of paramount importance to the well‐being of both humans and machines. Lattice structures, defined as architectured porous solids arranged in repeating patterns, are emerging as advanced sound‐absorbing materials. Their immense design freedom allows for customizable pore morphology and interconnectivity, enabling the design of specific absorption properties. Thus far, the sound absorption performance of various types of lattice structures are studied and they demonstrated favorable properties compared to conventional materials. Herein, this review gives a thorough overview on the current research status, and characterizations for lattice structures in terms of acoustics is proposed. Till date, there are four main sound absorption mechanisms associated with lattice structures. Despite their complexity, lattice structures can be accurately modelled using acoustical impedance models that focus on critical acoustical geometries. Four defining features: morphology, relative density, cell size, and number of cells, have significant influences on the acoustical geometries and hence sound wave dissipation within the lattice. Drawing upon their structural‐property relationships, a classification of lattice structures into three distinct types in terms of acoustics is proposed. It is proposed that future attentions can be placed on new design concepts, advanced materials selections, and multifunctionalities.

## Graphical abstract

Herein, the current progress, structural‐property relationships, underlying mechanisms, and offer insights into the future prospects of lattice structures is critically reviewed for sound absorption. Notably, novel classifications for sound‐absorbing lattices are being forwarded based on their structural characteristics and associated mechanisms. Building upon these, suggestions are put forth for future structural designs and propose innovative directions for further exploration.

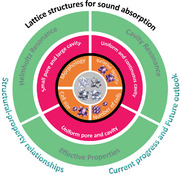

## 1 Introduction

Sound is an integral part of our daily lives. However, excessive or unwanted noise can be detrimental to the human well‐being. Noise pollution is a growing concern globally–where transportation, construction, and our daily lifestyles are major contributors. Apart from the human health, excessive noises are also detrimental to machines, in which they lead to overheating. Thus, sound absorbers are highly sought‐after in a variety of settings. For instance, theatres and rooms are installed with acoustic panels for sound absorption to reduce echoes. Many high‐power equipment is lined with sound absorbers to effectively attenuate excessive sound energy. Sound absorption is the process whereby sound energy is dissipated, and converted into heat, upon incidence onto a material. In an ideal situation, no waves are reflected off this surface. Common commercial sound absorbers include foams, fabrics, and microperforated panels. However, these materials have limitations such as weak absorption and limited bandwidth, and they lack the potential for multifunctionality.^[^ ^1^ ^]^ Modern engineering designs require advanced materials that are multifunctional and are optimized for mass and volume. As such, there is a growing need for new materials that offer superior acoustic properties and potential for customizable design optimization.

The advent of additive manufacturing has opened new opportunities for the design of functional materials based on structures rather than chemistry. Lattice structures are a new class of advanced material that manifests from this. Lattice structures are 3D structures composed of interconnected struts, shells, plates, or hybrids of these features, which form a repeating pattern.^[^ ^2^, ^3^ ^]^ These structures offer high degrees of design freedom for customizable feature‐pore morphology and interconnectivity, making them highly designable in achieving specific physical properties. Thus far, the physical properties of lattice structures have been widely studied and their strong potentials for applications as lightweight materials,^[^ ^4^ ^]^ energy absorbers,^[^ ^5^ ^]^ heat dissipater,^[^ ^6^ ^]^ heat insulator,^[^ ^7^ ^]^ and electromagnetic absorber/insulators,^[^ ^8^ ^]^ have been demonstrated. This implies that lattice structures have the potential to be customized to achieve specific acoustical properties. Thus far, the sound absorption properties of numerous types of lattice structures have been investigated. This includes lattice structures consisting of solely trusses,^[^ ^9^, ^10^, ^11^ ^]^ solely plates,^[^ ^11^ ^]^ trusses and plates,^[^ ^11^, ^12^ ^]^ tubes,^[^ ^13^, ^14^ ^]^ tubes and plates,^[^ ^15^, ^16^, ^17^ ^]^ smooth skeletal structures,^[^ ^18^, ^19^, ^20^ ^]^ triply periodic minimal surface,^[^ ^21^, ^22^ ^]^ and bioinspired features.^[^ ^23^ ^]^ Beyond the unit cell level, heterogeneities such as heterogeneously‐structured unit cells placed in parallel,^[^ ^12^, ^23^, ^24^ ^]^ functionally graded arrangements,^[^ ^25^, ^26^, ^27^, ^28^ ^]^ and additional acoustic meta‐features^[^ ^9^ ^]^ have also been proposed. Various unit cell sizes, feature sizes, and overall lattice thicknesses have been explored for these lattice structures. Like conventional sound absorbers, these structures exhibit unique sound absorption coefficient curves depending on their structural morphology. Additionally, lattice structures have proven to be favorable in comparison to conventional absorbers. This primarily attributes to them operating based on the same sound absorption principles, utilizing similar acoustical geometries, thus allowing them to exhibit comparable absorption characteristics. Furthermore, their extensive design flexibility offers additional opportunities for customization and optimization, enabling the attainment of superior performance. In addition to superior acoustic properties, lattice sound absorbers offer the potential for multifunctionality due to their unique physical properties, such as customizable or meta mechanical behaviors, which traditional materials lack. Works have shown that lattice structure sound absorbers also display the potential as lightweight high‐strength material,^[^ ^11^, ^16^, ^17^ ^]^ energy absorber,^[^ ^15^, ^23^ ^]^ and deformation‐recoverable materials.^[^ ^12^, ^24^ ^]^

Like traditional sound absorbers, the sound absorption properties of lattices are mainly geometry dependent. Through geometry, the acoustic impedance of lattice structures can be derived, and it can be further used to predict sound absorption properties. Thus far, various analytical models have been used to relate the lattice geometry to its acoustic impedance. Models include mechanisms based on multi‐layer Helmholtz resonance,^[^ ^11^, ^12^, ^14^, ^15^, ^16^, ^17^, ^23^, ^24^ ^]^ cavity resonance,^[^ ^13^ ^]^ effective acoustical properties,^[^ ^10^, ^25^ ^]^ and slow‐sound effects.^[^ ^9^ ^]^ These models have shown to produce high fidelity results in predicting sound absorption coefficients. They have thus also been used as reliable tools in optimizing lattice geometries for broadband absorption.^[^ ^12^, ^23^, ^24^ ^]^ Therefore, analytical acoustics models play a crucial role in facilitating the research of sound‐absorbing lattice structures.

As can be seen, research interest in lattice structures for sound absorption is on the rise. However, till date, no review of lattice structures for sound absorption has been conducted. A thorough background introduction to the fundamentals of sound absorption, overview of the current progress, an in‐depth analysis on structural‐property relationships, and the tools used for research would be valuable both as a lecture material to general readers and as a critical review for researchers working on lattice structures and acoustics. In this paper, we aim to provide such a detailed review on sound‐absorbing lattice structures. Additionally, we offer design recommendations and suggestions for future research on this topic toward the end of the review. This review is broken up into ten subsequent sections after this Introduction. Section 2 aims to lay the groundwork for sound absorption, Section 3 introduces the various classes of lattice structures, and Section 4 introduces the commonly used fabrication methods. Section 5 gives an overview on lattices studied for sound absorption, before going into the detailed analytical acoustics models used to model sound absorption properties in Section 6. Section 7 gives an overview of the finite element analysis simulation method to model absorption. Section 8 then takes into consideration all prior discussions and classifies lattices in accordance with their sound‐absorption mechanism and provides a detailed structural‐mechanism‐property relationship. Section 9 summarizes the multifunctionalities of sound‐absorbing lattice structures, apart from sound absorption. Section 10 provides recommendations on the design, future directions for the research on sound‐absorbing lattice structures, and brings forward some potential applications. Section 11 then concludes the review.

## 2 Principles of Sound Absorption

### 2.1 What is Sound Absorption?

**Figure 1:**
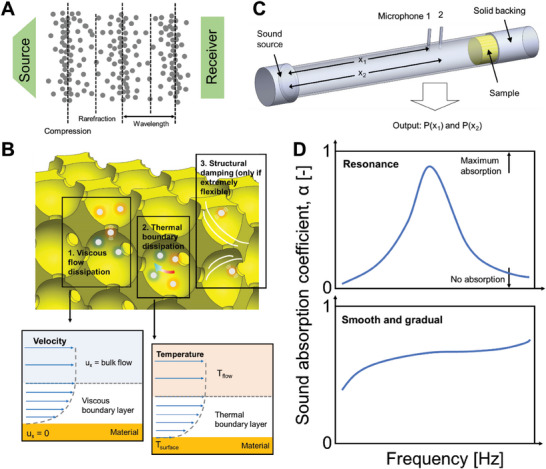
Fundamentals of sound absorption. A) Schematic of the principle of sound transmission through the air. B) Schematic of the sound dissipation mechanisms through a porous absorber, and illustration of the physics of the viscous flow and thermal boundary layers. C) Schematic of the sound absorption measurement using the impedance tube technique. D) Illustrations of two common types of sound absorption curves.

Sound absorption, as its name suggests, refers to the process by which a material absorbs sound energy as sound waves travel into it. As we know, sound waves are longitudinal waves. In the air, sound transmits through the motion of air molecules. This means that as opposed to the sound wave physically moving (unlike that of light), sound waves travel by the localized motion of air molecules (**Figure** **1**A). Specifically, the air molecules are the most compact at compression and most spread out at rarefraction. The wavelength and amplitude of this motion are then dependent on that of the sound source itself. The translation of this motion, whether by the cochlea in our ears or the diaphragm in microphones, is what converts it into signals that are further processed and perceived as “sounds.” Therefore, if the motions of the molecules are reduced, sound levels would be perceived to be reduced. Physically speaking, sound absorption refers to the conversion of the kinetic energy of the motion of molecules into thermal energy.

There are three main mechanisms for sound absorption. First, and also the most dominant mechanism, is dissipation at the viscous boundary layer.^[^ ^29^ ^]^ For any volume of air flowing over a material, a viscous boundary layer forms (Figure 1B). The viscous boundary layer forms as a transition region between the zero velocity over the surface of the material (no slip condition) and the bulk flowing air at the defined velocity. Thickness of the viscous boundary layer, δ_v_, is given by:^[^ ^30^ ^]^

(1) $$\delta_{v}=\sqrt{\frac{2\eta }{\rho \omega }}$$

η and ρ refer to the dynamic viscosity and density of air, respectively. ω=f refers to the angular frequency, with f being the frequency. The close proximity of air molecules to the material implies that they experience significant frictional forces when in contact with the material. The frictional forces thus reduce the extent of motion of the molecules. The most substantial contribution to sound absorption thus occurs through frictional dissipation at the viscous boundary layer. In terms of power loss per unit area, Q_v_, dissipation at the viscous boundary layer is calculated based on:^[^ ^31^ ^]^

(2) $$Q_{v}=\frac{1}{2}\;\eta \frac{v_{t}^{2}}{\delta_{v}}$$

*v*_t_ refers to the tangential velocity of air molecules across the viscous boundary layer and the other variables are listed in the nomenclature. During resonance, when air molecules vibrate most intensely, this frictional loss becomes notably amplified. As a result, sound absorption reaches its peak during resonance in the context of resonators.

Second, there is also thermal dissipation, arising from the thermal flux across the thermal boundary layer (Figure 1B).^[^ ^29^ ^]^ When sound waves propagate through a fluid medium, they create compressions and rarefactions that cause the fluid molecules to oscillate back and forth. This oscillation creates temperature variations within the fluid, which can lead to the formation of a thermal boundary layer. Thickness of the thermal boundary layer, δ_th_, is given by:^[^ ^30^ ^]^

(3) $$\delta_{th}=\sqrt{\frac{2\kappa }{\rho C_{p}\omega }}$$

κ and C_p_ refer to the thermal conductivity and heat capacity of the air, respectively. Heat transfer within the thermal boundary layer results in small‐scale fluid motion within its vicinity which thus generates frictional forces between adjacent fluid layers.^[^ ^29^ ^]^ Dissipation at the thermal boundary layer, *Q*_th_, in terms of power per unit area, translate to:^[^ ^31^ ^]^

(4) $$Q_{th}=\frac{1}{4}\;\left(\gamma -1\right)\frac{\omega p^{2}}{\rho c^{2}}\delta_{th}$$

γ refers to the ratio of specific heat and p refers to the pressure outside of the boundary layer. Thermal losses contribute minimally compared to viscous losses.^[^ ^32^ ^]^ Lastly, for materials which are extremely flexible or structures with high aspect ratios, dissipation contributed by physical vibration of the material/structure is not negligible. The vibration of the object causes it to physically disperse the air molecules, generating frictional forces that lead to the dissipation of sound energy. From the abovementioned mechanisms, it can be seen that sound absorbers should be either porous, so that sound waves can propagate into the pores, or are extremely soft, such that energy dispersion of sound waves is possible. Indeed, sound absorbers are mostly based on foams, porous panels, fabrics, or elastic materials and membranes currently.

### 2.2 Impedance Tube

The most widely adopted method for testing the sound absorption coefficients of materials is the impedance tube technique. An actual impedance tube, and a schematic of its working principle, is shown in Figure 1C. As its name suggests, the impedance tube is literally based on a tube. At one end, a loudspeaker fires off planar sound waves of desired intensity and frequency range. The sample to be tested lies at the other end. In line with the principle of sound absorption, e.g., the number of reflected waves off a rigid surface, the impedance tube includes a solid backing (dense solid such as steel, acrylic) immediately behind the sample. Following this, there are also two microphones used to collect the sound pressures at those respective locations. Signals collected by the microphones are then analyzed and converted into sound absorption curves. Herein, the transfer functions, based on that in the ASTM E1050‐19 standards, are adopted for the analysis. At any point in the tube, where A and B are constants, i is the imaginary unit and k is the wavenumber, the total sound pressure can be expressed as:

(5) $$P\;\left(x\right)=\;Ae^{-ikx}+Be^{ikx}$$

The sound pressures at microphones 1 and 2, at distances x_1_ and x_2_, respectively, from the sound source (Figure 1C), are then expressed as:

(6) $$P\;\left(x_{1}\right)=\;Ae^{-ikx_{1}}+Be^{ikx_{1}}$$

(7) $$P\;\left(x_{2}\right)=\;Ae^{-ikx_{2}}+Be^{ikx_{2}}$$

The transfer function between points 1 and 2 is calculated as:

(8) $$\;H_{12}=\;\frac{P\left(x_{2}\right)}{P\left(x_{1}\right)}$$

In terms of variables, the transfer function can also be expressed as:

(9) $$H_{12}=\frac{Ae^{-ikx_{2}}+Be^{ikx_{2}}}{Ae^{-ikx_{1}}+Be^{ikx_{1}}}$$

(10) $$H_{12}=\frac{e^{-ikx_{2}}+Re^{ikx_{2}}}{e^{-ikx_{1}}+Re^{ikx_{1}}}$$

$R\;=\frac{B}{A}\;$ is the reflection coefficient. R is solved using the current expression and the given and measured variables. Finally, the absorption coefficient is then calculated as:

(11) $$\alpha \;=\;1-{\left|R\right|}^{2}$$

Since k is frequency dependent, α is thus calculated at only one particular frequency for a given k. The whole sound absorption curve is obtained across the range of frequencies.

### 2.3 Sound Absorption Coefficient Curve

Examples of sound absorption coefficient curves are shown in Figure 1D. The y‐axis corresponds to the intensity of absorption, ranging from 0 to 1, with 1 referring to the highest absorption. The x‐axis corresponds to the frequency. Different types of sound absorption coefficient curves exist. One commonly observed type is those with one (or more) absorption resonance peak. This type of curve is characterized by high coefficients nearing unity but with however a narrow bandwidth. Such properties are usually found in perforated panels and structural metamaterial absorbers. Another type is those with a moderate intensity of absorption, but with a flat or gradient curve across the entire frequency range. This type is in turn commonly observed in open‐cell foams, fabrics, or any fibrous material. Combination of different features of the sound absorbers may result in absorption curves of a hybrid morphology. Nonetheless, it is intuitive that the higher the curve, and the broader it is, the better the material is at sound absorption. However, sound absorption coefficient curves are highly dependent on the thickness of the sample. In general, higher absorption is achieved with thicker samples. However, when it comes to practical product design aimed at maximizing performance efficiency, our goal is to minimize both the mass and volume as much as possible.

### 2.4 Acoustic Impedance

The specific acoustic impedance, Z, refers to the opposition of the system, to the acoustic flow, that results from the acoustical pressure being applied to it. Herein, the system refers to the structure of the sound‐absorbing material. This is analogous to electrical impedance, which refers to opposition to the electrical current that flows from the applied voltage. Similarly, Z is a complex number consisting of the real part, which refers to the acoustic resistance, and the imaginary part, which refers to the mass reactance. Physically speaking, the real part describes the extent of energy loss in the system and the imaginary part determines the frequency at which resonance occurs.^[^ ^31^ ^]^ *Z* is closely related to the reflection coefficient (*R*) via the following relationships:

(12) $$R\;=\;\frac{Z/Z_{0}-1}{Z/Z_{0}+1}$$

(13) $$Z\;=\;Z_{0}\left(\frac{1+R}{1-R}\right)$$

Z_0_ refers to the acoustic impedance of air at standard conditions, given by the multiplication of the speed of sound, c_0_, and density, ρ_0_, of air at standard conditions. The relative acoustic impedance, *Z*_r_, is then expressed as:

(14) $$\;Z_{r}=\frac{Z}{Z_{0}}\;$$

As will be detailed in the later sections, it is the effective *Z*_r_ of the sound‐absorbing structures that we are trying to derive in the analytical models. With *Z*_r_, we can then derive the absorption coefficient curve. The absorption coefficient, α, is calculated through the real (Re) and imaginary (Im) components of *Z*_r_ via:

(15) $$\alpha \;=\frac{4Re\left(Z_{r}\right)}{{\left[1+Re\left(Z_{r}\right)\right]}^{2}+Im{\left(Z_{r}\right)}^{2}}$$

In particular, to achieve ideal absorption, we aim for impedance matching. For maximum absorption to occur, e.g. where α = 1, Re(*Z*_r_) should be ideally 1 while Im(*Z*_r_) should be ideally 0. Impedance matching is attained in such a scenario.

## 3 Lattice Structures

Lattice structures are defined as periodic and repeating unit cells consisting of 3D spatially architectured features. For its non‐rigorous definition, lattice structures can thus incorporate any types of features and with virtually unlimited design possibilities. Here, in terms of the physical appearance, we categorize lattice structures into three primary classes: truss, triply periodic minimal surface (TPMS), and plate, with a fourth category comprising hybrid features of these structures.

### 3.1 Truss Lattices

**Figure 2:**
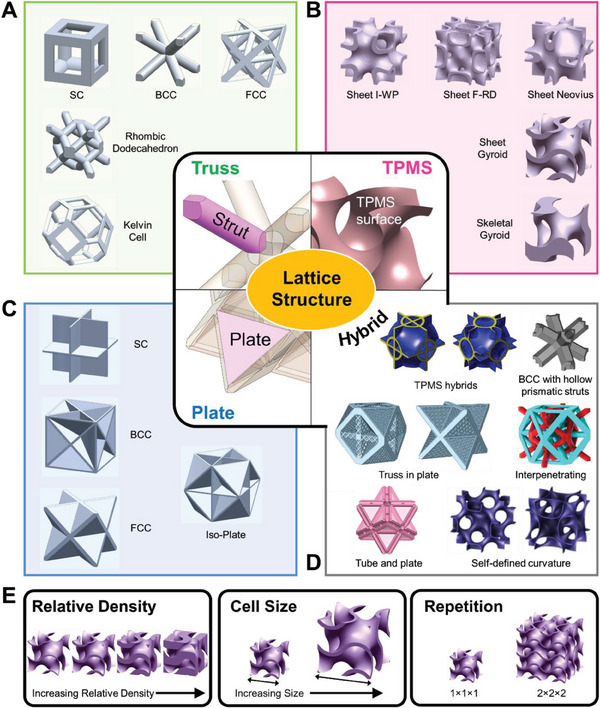
An overview of the types of lattice structures. The fundamental features and examples of unit cells of A) truss, B) TPMS, C) plate, and D) hybrid lattice structures. For the hybrid structures, references used are as follows. TPMS hybrids: Reproduced with permission.^[^ ^59^ ^]^ Copyright 2019, Elsevier. BCC with hollow prismatic struts: Reproduced with permission.^[^ ^52^ ^]^ Copyright 2023, Elsevier. Truss in plate: Reproduced with permission.^[^ ^61^ ^]^ Copyright 2023, Wiley. Interpenetrating: Reproduced with permission.^[^ ^64^ ^]^ Copyright 2021, Elsevier. Tube and plate: Reproduced with permission.^[^ ^16^ ^]^ Copyright 2023, American Chemical Society. Self‐defined curvature: Reproduced with permission.^[^ ^56^ ^]^ Copyright 2019, Elsevier. E) Illustrations of the other characteristics of lattices, including the relative density, cell size, and repetition.

Like their architectural counterparts, truss lattices consist of interconnected struts joint at nodes (**Figure** **2**A). The strut orientation, the number of struts, and the number of nodes are the main characteristics that define the truss lattice structure. The simplicity of the design motifs in truss lattices allows for significant design freedoms, resulting in virtually unlimited design possibilities. Some of the fundamental and highly studied designs are associated with the mimicry of Bravais lattices, such as the simple cubic (SC), body‐centred cubic (BCC), and face‐centred cubic (FCC) crystal structures. Permutations of these crystals to achieve specific physical properties also exist.^[^ ^33^, ^34^, ^35^ ^]^ Truss lattices based on other geometries also include those based on biological structures,^[^ ^36^, ^37^, ^38^ ^]^ polyhedrons,^[^ ^39^, ^40^ ^]^ and covalent bonds.^[^ ^40^ ^]^ Otherwise, truss lattices can also be generated through topological optimization using a finite element software.^[^ ^41^, ^42^ ^]^ Most of the times, struts are designed to be uniform. However, there are also truss lattices with struts designed to be tapered struts or of heterogeneous cross sections for customized mechanical properties.^[^ ^34^, ^43^ ^]^

### 3.2 TPMS Lattices

Triply periodic minimal surface (TPMS) lattices are structures with geometry defined using TPMS surfaces (Figure 2B). TPMS surfaces are 3D mathematical surfaces with non‐intersecting parts and zero mean curvature. They are first introduced by Hermann Schwarz in 1865, and then further developed by Alan Schoen in 1970 where more TPMS surfaces were introduced. Some of the most common TPMS lattices include the Gyroid, Diamond, Primitive, I‐WP, Neovius, and F‐RD.^[^ ^44^ ^]^ Each type of TPMS surface is essentially described by its own parametric equation, for instance, the gyroid is based on: sin (x)cos (*y*) + sin (y)cos (*z*) +  sin (z)cos (*x*) =  t, where t = 0. The most fundamental TPMS lattices are those based on the thickened mathematical surface, where they are also known as sheet TPMS. As such, sheet TPMS are sometimes also known as shell lattices for their smooth curvature. Since they are continuous, another form of TPMS lattice is based on the inverse of the volume taken up by the thickened sheet.^[^ ^45^ ^]^ This then constitutes skeletal TPMS. As opposed to shells, skeletal TPMS lattices look more alike to truss lattices that are composed of smooth struts. With aims of achieving enhanced direction strength or isotropy, variations of TPMS lattices based on modified parametric equations exist. These include those with modified parameter t,^[^ ^46^ ^]^ and those with varying wall thicknesses.^[^ ^47^ ^]^

### 3.3 Plate Lattices

Plate lattices are in turn a newer concept proposed in recent years. Analogous to truss lattices, plate lattices consist of plates arranged strategically (Figure 2C). For instance, Bravais lattice based plates lattices consist of plates being positioned at close‐pack planes of the respective crystal structures.^[^ ^4^ ^]^ The first plate lattice is proposed by Berger et al., where the authors also revealed that a structure consisting of a combination of SC+FCC plates is capable of reaching the Hashin‐Shtrikman upper bounds for isotropic elasticity.^[^ ^48^ ^]^ However, plate lattices based on Bravais lattices have a closed‐cell morphology, rendering them to be incapable of being manufactured by many 3D printing techniques. To address this challenge, researchers have introduced “semi‐plate” lattices, where the plates are implemented with strategically positioned pores to facilitate the removal of feedstock material that might otherwise become trapped within closed cells.^[^ ^4^, ^11^, ^49^ ^]^ Apart from these, researchers have also proposed novel plate lattices with plate arrangements that form open‐cells.^[^ ^50^, ^51^ ^]^

### 3.4 Hybrid Structures

Struts, shells, and plates are the three most common types of features observed in lattice structures. Nonetheless, various works have reported modifications or hybrid combinations of these features (Figure 2D), in search for novel lattices with unique properties. One popular class is a variant of truss lattices–tube lattices–where the struts are made hollow instead of being solid. Lattices of both cylindrical and prismatic tubes have been reported.^[^ ^38^, ^52^, ^53^ ^]^ Other popular types also include shell lattices defined based on other novel mathematical definitions and criteria, apart from the TPMS criteria.^[^ ^54^, ^55^, ^56^ ^]^ In terms of hybridization and combination, combinations of shells and tubes,^[^ ^57^ ^]^ tubes and plates,^[^ ^58^ ^]^ TPMS and plates,^[^ ^59^ ^]^ TPMS and struts,^[^ ^60^ ^]^ struts and plates,^[^ ^11^, ^61^, ^62^ ^]^ interpenetrating lattices,^[^ ^63^, ^64^ ^]^ exist. In addition to occurring at the unit cell level, hybridization also occurs when arranging multiple unit cells. For instance, the repeating lattice consists of unit cells that are different from one another. These include the functionally graded,^[^ ^65^ ^]^ fractal,^[^ ^66^ ^]^ hierarchical,^[^ ^67^, ^68^ ^]^ and heterogeneous^[^ ^69^ ^]^ types of arrangements.

### 3.5 Classification of Lattice Structures

Despite the virtually unlimited possibilities with the design of lattices, there are only two intrinsic factors sufficient in defining a lattice structure. First, the lattice morphology, and second, the relative density. The lattice morphology pertains to the distinctive structures that lattices can adopt, and they have been elucidated in Sections 3.1–3.4. The relative density then refers to the volume fraction of the solid parts of the lattice structure with respect to the volume of its bounding box. In other words, lattices with a higher relative density have thicker struts, shells, or cell walls, etc (Figure 2E). The unit cell size then falls under as an extrinsic factor, e.g. increased unit cell size only results in a proportional scaling of all features in a lattice (Figure 2E). A final characteristic is the cell repetition, which refers to any number of times a unit cell, either the same one or a different one, is repeated across any direction to form a repeating lattice (Figure 2E).

## 4 Fabrication of Lattice Structures

The intricate geometry required for lattice structures to achieve effective sound absorption has limited their manufacturing to primarily additive manufacturing (AM) techniques. Specifically, AM processes that enable high levels of geometrical complexity, including vat photopolymerization and powder‐bed fusion based techniques, as well as fused deposition modelling, have thus far been adopted for this purpose.

### 4.1 Vat Photopolymerization

Vat‐photopolymerization techniques involve the polymerization of photosensitive resin. The most commonly used setup is a top‐down building approach, consisting of a building substrate, light source, and a resin vat with an optically transparent film. The main printing process involves i) the substrate being lowered into the vat, ii) the light source patterning onto the resin layer sandwiched between the film and substrate, which thereby solidifies the photosensitive resin, iii) the substrate pulling up from the vat, and iv) the process repeating until the desired 3D solid is formed. Two main types of vat‐photopolymerization techniques exist: digital light processing (DLP) and stereolithography (SLA). These techniques function on the same basis with the only difference being the type of light source used. DLP uses a digitally projected light while SLA uses a scanning laser. Despite the different types of light sources, light energies generally fall within the UV‐visible spectrum with 380–405 nm wavelength. Proper selection of light energy and layer thickness, where they should be matching, is critical to successful builds. Insufficient light energy density results in non‐forming while excessive results in the resin decomposition. Owing to the highly customizable chemical composition of the resin, printed solid polymer of a variety of mechanical properties can be achieved.^[^ ^70^ ^]^ Apart from pure polymers, resin‐derived ceramics and metals are also possible using vat photopolymerization.^[^ ^71^ ^]^

### 4.2 Powder‐Bed Fusion

The powder‐bed fusion (PBF) technique is a type of AM technique that involves the fusion of powders in a powder bed to create solid objects. PBF involves three main components: the building substrate, energy source, and powder feeding system. The main printing process involves i) a layer of feedstock material powder being laid onto the building platform, ii) an energy source melts/sinters the powder into a solid entity, and the iii) process repeats itself until the desired 3D structure is obtained. Main parameters in a PBF process include the energy source power, scan speed, hatch distance, and layer thickness. Different PBF techniques differ in their energy sources and fusion mechanisms. The three most common PBF techniques are selective laser melting (SLM), electron beam melting (EBM), and selective laser sintering (SLS). Their differences lie mainly in their energy source and the materials suitable for fabrication. SLM uses high‐intensity lasers, EBM uses electrons, and SLS uses a low‐intensity laser. Metals, such as steel, titanium, nickel, aluminium alloys, are printed through SLM and EBM^[^ ^72^ ^]^ while thermoplastics, such as polyamide, polystyrene, are better printed through SLS.^[^ ^73^ ^]^ Optimal parameters for PBF processes need to be established for individual materials such that parts can be printed near net densities and with the required mechanical properties.

### 4.3 Fused Deposition Modelling

Fused deposition modelling (FDM) belongs to the class of extrusion‐based AM technique and is based on the principle of extruding a material melt. FDM utilizes a filament as its feedstock. The filament is then melted using a heating mechanism, typically a nozzle, and then deposited onto a substrate, just like how a pen writes on a piece of paper. Common FDM filaments include pure thermoplastics such as polylactic acid and acrylonitrile butadiene styrene, and thermoplastics composites (with metal powder, carbon fibre, etc).^[^ ^74^ ^]^ Owing to its printing process, FDM has the advantage of leaving no residual feedstock materials after printing, which allows for the creation of structures with completely closed cells.

## 5 Overview on the Sound Absorption of Lattice Structures

**Figure 3:**
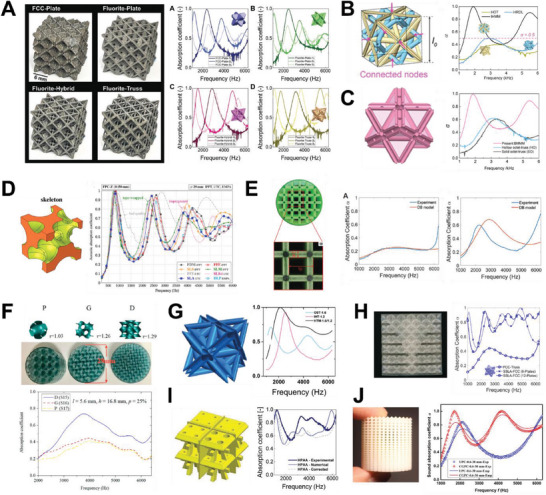
An overview of the current research on lattice structures for sound absorption, including illustrations of their structure and typical sound absorption curves. Lattices with absorption curves consisting of distinct resonance peaks. A) Illustrations of 3D printed metal FCC‐based truss and plate lattices and their sound absorption curves. Reproduced with permission.^[^ ^11^ ^]^ Copyright 2021, Wiley. B) Illustration of an interpenetrating lattice, and the sound absorption curves of the interpenetrating lattice and its constituents. Reproduced with permission.^[^ ^14^ ^]^ Copyright 2023, American Chemical Society. C) Illustration of a bamboo‐inspired lattice, and the sound absorption curves of the bamboo‐inspired lattice and its solid truss and hollow truss counterparts. Reproduced with permission.^[^ ^16^ ^]^ Copyright 2023, American Chemical Society. D) Illustrations of pore and cavity lattices and their sound absorption curves. Reproduced with permission.^[^ ^18^ ^]^ Copyright 2020, Elsevier. E) Illustration of a simple cubic lattice, and the sound absorption curves of its low relative density and high relative density structures. Reproduced with permission.^[^ ^10^ ^]^ Copyright 2022, Whoice Publishing. F) Illustration of primitive, gyroid, and diamond TPMS lattices and their sound absorption curves. Reproduced with permission.^[^ ^21^ ^]^ Copyright 2020, Taylor & Francis. G) Illustration of a hollow truss lattice, and the sound absorption curves of the hollow truss and its constituent phases. Reproduced with permission.^[^ ^13^ ^]^ Copyright 2022, Wiley. H) Illustration and the sound absorption curves of a truss lattice and its sonic blackhole composites. Reproduced with permission.^[^ ^9^ ^]^ Copyright 2023, Elsevier. I) Illustration of a parallel heterogeneous lattice structure and its sound absorption curve. Reproduced with permission.^[^ ^12^ ^]^ Copyright 2021, Wiley. J) Illustration of a functionally graded truss lattice, and the sound absorption curves of the functionally graded truss and its uniform counterpart. Reproduced with permission.^[^ ^27^ ^]^ Copyright 2019, Elsevier.

Thus far, the sound absorption properties of almost all types of lattice structures that were mentioned in Section 3 have been investigated. This includes fundamental truss structures like the SC, BCC, FCC,^[^ ^9^, ^10^ ^]^ plate lattices based on Bravais lattices,^[^ ^11^ ^]^ hybrid truss and plate lattices,^[^ ^12^ ^]^ hybrid tube lattices,^[^ ^13^ ^]^ hybrid tube and plate lattices,^[^ ^15^, ^16^, ^17^ ^]^ hybrid truss and smooth shell lattices,^[^ ^18^, ^19^, ^20^ ^]^ TPMS structures,^[^ ^21^ ^]^ bioinspired structures,^[^ ^23^ ^]^ and functionally graded trusses.^[^ ^25^, ^26^, ^27^, ^28^ ^]^ Also, a diverse range of cell sizes and lattice thickness were investigated. In general, unit cell sizes studied ranges from anywhere up to 10 mm. In turn, thickness, depending on both the unit cell size and the number of layers, ranges from around 20 up to 60 mm. For these geometries, the frequency range of interest for lattice structures generally lies from around 1000 to 6000 Hz. Like conventional sound absorbers, lattice structures exhibit different types of sound absorption coefficient curves based on their unique structural morphology. Also, lattice structures have shown the capability to produce all the types of absorption curves that were depicted in Section 2.3, and they compare favorably to commercial absorbers. For instance, **Figure** **3**A–D depicts the sound absorption curve of lattices with distinctive resonant peaks, akin to perforated panels. Figure 3E,F then shows absorption curves that are smooth and gradual, more foam and fabric like. The primary reason for this is attributed to their functioning based on the exact same principle as traditional materials. In other words, mechanism‐guided structural optimization has also been adopted for the design of these lattices. This thus constitutes heterogeneous lattices, either in terms of mechanisms, or structures. Thus far, lattices with dual sound dissipation mechanisms include hollow trusses (Figure 3G) and sonic black hole lattices (Figure 3H). In terms of structures, heterogeneous assembly of unit cells (Figure 3I) and functionally graded structures (Figure 3J) have been worked on. Improved sound absorption, either in terms of increased bandwidth or the intensity of absorption, has been observed in these heterogeneous lattices as compared to their constituents. Introducing heterogeneity is thus an effective way to improve sound absorption while maintaining sample thickness.

Like any sound absorber, sound absorption is mainly dependent on the geometry of the lattice. This implies that its acoustic impedance can be calculated using its geometry, which can, in turn, be utilized to predict its sound absorption properties. Therefore, Section 6 is dedicated to discussing the various analytical acoustics models that have thus far been used for modelling sound absorption in lattice structures. Section 8 then gives a thorough overview of the structure‐mechanism‐property relationship on lattice structures for sound absorption. Apart from their good sound absorption capabilities, lattice structures are also advantageous to commercial absorbers as they can multi‐function as lightweight materials or energy absorbers. This is then illustrated in Section 9.

## 6 Analytical Acoustics Models

### 6.1 The Transfer Matrix Method

#### 6.1.1 Series

**Figure 4:**
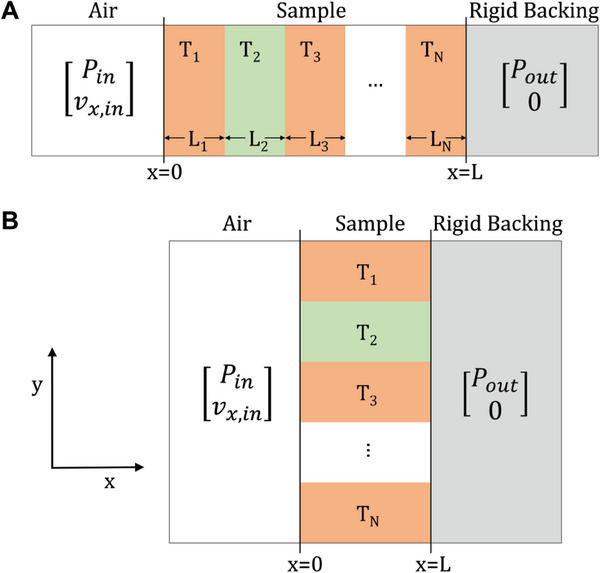
Schematic of the transfer matrix calculations for modelling the effective acoustic impedance for multi‐feature sound absorbers under the rigid backing condition. The A) case for multi‐layered absorber in series and the B) case for absorbers with heterogeneous features in parallel.

As mentioned, sound wave energy dissipation depends solely on the shape of the air bound by the material of the cellular solid. Where necessary, this shape (or also the shape of the cellular solid) can be regarded as alternating and periodic layers of different structures. One implication of this morphology is that the overall acoustic impedance of the structure needs to be a summation of the acoustic impedance of each type of layer, along the direction of the wave propagation. The transfer matrix method (TMM) is a powerful tool capable of carrying out this operation. Physically, the TMM methods links the acoustic pressure, P, and the normal velocity, v_x,in_, on the inlet (x = 0) and the outlet (x = L) of any sound‐absorbing material of thickness L with a rigid solid‐backing, via the following relationship (**Figure** **4**A):

(16) $$\begin{array}{c} \;{\left[\begin{array}{c} P_{in}\\ v_{x,in} \end{array}\right]}_{x\;=\;0}=\;T{\left[\begin{array}{c} P_{out}\\ 0 \end{array}\right]}_{x\;=\;L} \end{array}$$

***T*** refers to the 2 × 2 transfer matrix of the sound‐absorbing material. The expressions for each of its four terms is highly dependent on the microstructure of the material and they take on different forms. This will be detailed in the later sub‐sections. For a single material of homogeneous microstructure (such as foams), ***T*** refers to the transfer matrix of the material. Since lattice structures usually consist of multiple layers of acoustically distinct microstructure, a transfer through all these layers is required to derive ***T*** instead. For a material of N layers, each of thickness of *L*_i_, where ***T*_i_** is the transfer matrix of a layer, ***T*** can be given as:

(17) $$\begin{array}{c} T\;=\prod_{i=1}^{N}T_{i}\;=\left[\begin{array}{cc} T_{11} & T_{12}\\ T_{21} & T_{22} \end{array}\right]\; \end{array}$$

The specific acoustic impedance, *Z*, of the material is calculated from the overall matrix, ***T***, by:

(18) $$Z\;=\frac{T_{11}/T_{21}}{Z_{0}}\;$$

#### 6.1.2 Parallel

Apart from multilayered structures, the TMM can also be used to model sound absorbers with parallel arranged heterogeneous components. For instance, shown in Figure 4B, the components are placed side‐by‐side now, as opposed to being stacked along the direction of the sound wave. This method is first proposed by Verdiere et al.^[^ ^75^ ^]^ For convenience in the mathematical expressions, we first convert the transfer matrices of each individual component (**T_i_**) into their admittance matrices, **Y_i_**:

(19) $$\begin{array}{c} \;\left[\begin{array}{c} v_{x,i}\\ v_{x,i}^{\prime } \end{array}\right]=Y_{i}\;\left[\begin{array}{c} P_{i}\\ P_{i}^{\prime } \end{array}\right] \end{array}$$

(20) $$\begin{array}{c} \;Y_{i}=\left[\begin{array}{cc} Y_{i,11} & Y_{i,12}\\ Y_{i,21} & Y_{i,22} \end{array}\right]\;=\frac{1}{T_{i,12}}\;\left[\begin{array}{cc} T_{i,22} & T_{i,12}T_{i,12}-T_{i,22}T_{i,11}\\ 1 & -T_{i,11} \end{array}\right] \end{array}$$

With the admittance matrices of each component obtained, the overall transfer matrix for the parallel case, **T_P_**, can be calculated via the following transformation:

(21) $$\begin{array}{ccc} T_{P} & = & \frac{-1}{\sum r_{i}Y_{i,21}}\;\;\left(\begin{array}{cc} \sum r_{i}Y_{i,22} & -1\\ \sum r_{i}Y_{i,22}\sum r_{i}Y_{i,11}-\sum r_{i}Y_{i,12}\sum r_{i}Y_{i,21} & -\sum r_{i}Y_{i,11} \end{array}\right)\\ & = & \left[\begin{array}{cc} T_{p,11} & T_{p,12}\\ T_{p,21} & T_{p,22} \end{array}\right] \end{array}$$

r_i_ is the area fraction of the i^th^ component with respect to that of the entire material.

### 6.2 Helmholtz Resonance

#### 6.2.1 Multilayered Helmholtz Resonator Model

**Figure 5:**
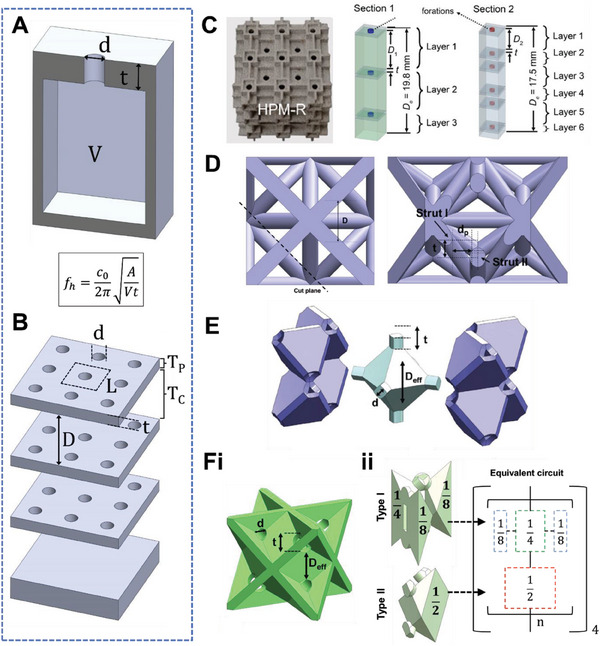
On the Helmholtz resonance mechanism and MLHR‐based lattice structures. A) The cross sectional view of a typical Helmholtz resonator and key acoustical geometries influencing its resonant frequency. B) The morphology of a MLHR, or MPP, and illustrations of the key acoustical geometries influencing its resonant frequency, and the transfer matrices. C) Illustrations of a cubic plate lattice with unit that resembles a Helmholtz resonator, and how the discretization of its layers to be modelled as an MLHR. Reproduced with permission.^[^ ^17^ ^]^ Copyright 2022, Taylor & Francis. D) Illustrations of the Helmholtz acoustical geometries, from the cross sectional view, of a truss lattice. Reproduced with permission.^[^ ^13^ ^]^ Copyright 2022, Wiley. E) Illustrations of the Helmholtz acoustical geometries of a plate lattice with pores. F) For a complex plate lattice, illustration of the i) Helmholtz acoustical geometries of the unit cell, and ii) discretization of the lattice into two types of layers, revealing its representative equivalent circuit. Reproduced with permission.^[^ ^11^ ^]^ Copyright 2021, Wiley.

Thus far, most of the sound‐absorbing lattice structures function based on Helmholtz resonance. Named after the German physicist Hermann von Helmholtz, a Helmholtz resonator is a bottle‐like enclosure, based on a narrow neck, followed immediately by a notably large cavity. The Helmholtz resonator operates on the principle of trapping and amplifying specific frequencies of sound by utilizing a cavity and a neck. The cross sectional view of a cuboid Helmholtz resonator is shown in **Figure** **5**A. There are three acoustical geometrical parameters of concern, namely the surface area of the pore, A, thickness of the neck, t, and the volume of the cavity, V. Collectively, the resonant frequency of the Helmholtz resonator, f_h_, can be calculated using these parameters through the following relationship:

(22) $$\;f_{h}=\frac{c_{0}}{2\pi }\;\sqrt{\frac{A}{Vt}}$$

At resonance, the air oscillates most vigorously at the neck of the Helmholtz resonator. A highly concentrated thermal and viscous dissipation thus takes place there. In 1998, Maa expanded upon the concept of Helmholtz resonators by introducing the concept of microperforated panels (MPP).^[^ ^76^ ^]^ These panels, essentially large cavities with multiple pores, can be considered to be a specific type of Helmholtz resonator. Typically, MPPs are constructed using multiple layers, forming a configuration known as a multi‐layered Helmholtz resonator (MLHR). The schematic of an MPP is shown in Figure 5A. As seen, an MPP consists of a rigid panel, with numerous pores, or holes, introduced, and is being backed by an open cavity of air. Herein, the geometrical parameters of concern include the pore diameter, d, pore thickness, t, depth of the air‐backed cavity, D, and the surface porosity, σ. The surface porosity, also known as the perforation ratio, is the ratio of the surface area of a pore, to a unit area of the panel. For an MPP with pore diameter d and a unit panel of length L (Figure 5B), σ  = [π(*d*/2)^2^]/*L* ^2^ . Up to this step, it is clear that the narrow pore and open‐air cavity of an MPP is reminiscent of the structure of a Helmholtz resonator. Indeed, for a single‐layered MPP, its resonant frequency is equivalent to f_h_ calculated using Equation (22).^[^ ^24^ ^]^ However, MLHRs are more sought‐after for practical applications due to their ability to introduce a greater number of resonant peaks, resulting in a broader absorption bandwidth.

Interestingly, many lattice structures take up the morphology of an MLHR. This also implies that these lattice structures can be sufficiently described using the geometrical parameters d, t, D, and σ, for elucidating their sound absorption properties. As noted from Section 6.1, the overall acoustic impedance of a material can be derived using TMM. For MLHRs, there are two distinct sets of transfer matrices and corresponding acoustic impedances — one linked to the narrow pore and the other to the large cavity. We first devise the acoustic impedance of the narrow pore. The acoustic impedance through a narrow pore is given as:

(23) $$Z_{P}=\;i\omega \rho t{\left[1-\frac{2}{k\sqrt{-i}}\frac{J_{1}\left(k\sqrt{-i}\right)}{J_{0}\left(k\sqrt{-i}\right)}\right]}^{-1}$$

J_0_ and J_1_ represent the zeroth and first‐order Bessel functions, respectively. In the configuration of an MLHR, where there are numerous pores, Z_p_ needs to be normalized by the area ratio taken up by the pores:

(24) $$\;Z_{P}=\frac{Z_{P}}{\sigma }\;$$

Avoiding the complex Bessel function calculations, one simpler version of Equation (23) can be expressed as follows:^[^ ^76^ ^]^

(25) $$\;Z_{P}=\frac{32\eta t}{d^{2}\sigma }\;\left(\sqrt{1+\frac{k_{d}^{2}}{32}}\right)+i\frac{\omega \rho t}{\sigma }\left(1+{\left(9+\frac{k_{d}^{2}}{2}\right)}^{-\frac{1}{2}}\right)$$

$\;k_{d}=\;d\sqrt{\rho \omega /4\eta }$ refers to the perforation constant. In Equation (25), the real part refers to the acoustic resistance and the imaginary part refers to the mass reactance of the perforated surface. When the pore diameter and thickness are smaller than the wavelength of the propagating sound, the volume of resonating air travels beyond the thickness of the pore. This additional air mass needs to be accounted for. Further, for the complex pore and cavity architecture in many MPPs and lattice structures, Equation (25) is insufficient to represent the actual sound wave dissipation through the pore. Therefore, for most of the cases, end corrections to both the real and imaginary parts are necessary. The former accounts for the air friction formed at the surface of the panel when the air propagates through the narrow pore while the latter accounts for the oscillatory motion extended beyond the thickness of the pore. One common form of Equation (25), with end correction terms, is expressed as:^[^ ^12^, ^76^, ^77^ ^]^

(26) $$\begin{array}{ccc} Z_{P} & = & \frac{32\eta t}{d^{2}\sigma }\;\left(\sqrt{1+\frac{k^{2}}{32}}+2\varepsilon R_{s}\right)\\ & & +i\frac{\omega \rho_{0}t}{\sigma }\left(1+{\left(9+\frac{k^{2}}{2}\right)}^{-\frac{1}{2}}+\delta \frac{d}{t}\right) \end{array}$$

As opposed to being scientifically derived, end corrections are usually obtained empirically or semi‐empirically based on experimental data. The end correction for the air resistance includes variables ε and $R_{s}=\sqrt{2\eta \rho_{0}\omega }/2$. ε is mainly geometry dependent on the pore shape, thickness, and surface finish quality, and it is usually derived empirically through experiments. Various ε values have been proposed by researchers. ε has been proposed originally as 0.5 by Maa.^[^ ^76^ ^]^ Later on, Guo et al. suggested that ε can be either 2 and 4, for a rounded and sharp‐edged pore finish, respectively.^[^ ^78^ ^]^ Bolton et al. instead proposed a frequency dependent ε through computational fluid dynamics.^[^ ^79^ ^]^ The end correction term for mass reactance includes a variable δ and the ratio of the geometrical parameters d and t. Similarly, numerous versions of the mass reactance end correction have been reported. In general, it is geometry and size dependent. Ingard suggested that a universal correction term for any geometry can be given as $0.96\sqrt{A}/t$, where A refers to the area of the pore.^[^ ^80^ ^]^ For the special case of a circular pore, A then corresponds to π(*d*/2)^2^ and the end correction term simplifies to 0.85*d*/*t*, where δ equals to 0.85. This end correction is widely used by researchers for standard MPPs. Additionally, Ingard also suggested that δ should decrease in value with increasing surface porosity. Apart from these, other researchers also proposed σ dependent δ for square pores.^[^ ^81^ ^]^ Recent studies also suggest that both ε and δ can be geometry dependent too, in which they should increase as the cavity wall get increasingly close to the pore.^[^ ^23^, ^82^ ^]^ All in all, the end correction terms are highly dependent on the geometry of the multi‐layered Helmholtz resonator, case‐by‐case. Having the acoustic impedance of the pore, Z_P_, derived, the transfer matrix of the pore layer is then given as:^[^ ^83^ ^]^

(27) $$\;T_{P}=\left[\begin{array}{cc} 1 & Z_{P}\\ 0 & 1 \end{array}\right]\;$$

The acoustic impedance of the cavity is then dependent on the cavity depth, D, solely. Straightforward, the transfer matrix of the cavity, T_C_, is given as:^[^ ^83^ ^]^

(28) $$\;T_{C}=\left[\begin{array}{cc} \cos\left(k_{0}D\right) & iZ_{0}\sin\left(k_{0}D\right)\\ \frac{i\sin\left(k_{0}D\right)}{Z_{0}} & \cos\left(k_{0}D\right) \end{array}\right]\;$$

For MPPs and lattices with cavities without significant obstructing features, such as struts, or cavities which take on a unique geometry, D refers to the distance between the bottom and top of successive pores. Otherwise, correction factors may be required. Li et al.^[^ ^11^ ^]^ and Cheng et al.^[^ ^77^ ^]^ suggested that the D for octahedral and spherical cavities should be based on the length of a cube of an equivalent volume to the shape. Li et al. also suggested that if a significant volume of struts exist in the cavity, the reduction in the free space should be accounted for by the reduction in D.^[^ ^24^ ^]^

Therefore, for a unit MLHR consisting of a pore and cavity, the overall matrix, **T_T_**, is (Figure 5B):

(29) $$\;T_{T}=T_{P}\;\cdot T_{C}$$

For N number of layers, **T_T_** is simply (Figure 4A):

(30) $$\;T_{T}=\prod_{i=1}^{N}T_{i}\;=T_{P1}\;\cdot T_{C1}\cdot T_{P2}\cdot T_{C2\;}\cdots \;T_{PN}\cdot T_{CN}$$

It is to note that for each layer, variables d, t, D and σ can take on different values. This then constitutes the concept of heterogeneously structured MLHRs.

#### 6.2.2 Heterogeneous Case

A homogeneous Helmholtz resonator is only associated with limited resonance peaks (one or more depending on the harmonics) related to its acoustical geometry. These peaks are narrow, and these absorbers thus do not display broadband absorption behaviors. Instead, enhanced absorption bandwidth can be achieved via introducing structural heterogeneity, either i) along (in series) or ii) normal (parallel) to the direction of the sound wave propagation. In essence, the underlying principle is that resonance peaks at different frequencies work in tandem and the resulting absorption curve is a superposition of the individual peaks. To achieve heterogeneity in series, designers can simply assign different [d, t, D and σ] acoustical geometries to each layer. The overall transfer matrix through the layers (and therefore acoustic impedance) can be derived by subbing in the respective values into Equation (30). Different from the series case, the overall acoustic impedance of heterogeneous cells assembled in parallel is summed up through the parallel rule (analogous to the mixture‐of‐composites rule):

(31) $$Z\;=\frac{A}{\sum_{i=1}^{4}\frac{A_{i}}{Z_{i,1}}}\;$$

A is the overall surface area of the heterogeneous cells, A_i_ and Z_i_ are the surface area and the acoustic impedance of each individual cell, respectively. To achieve this, different resonant cells must be fully partitioned where no lateral sound wave propagation is possible. Alternatively, TMM summation in parallel, as described in Section 6.1.2, can be considered. Improved bandwidths of absorption upon heterogeneous designs have been observed for all cases in literature.^[^ ^11^, ^12^, ^17^, ^23^, ^24^ ^]^

#### 6.2.3 Examples

Various works suggested that lattice structures can be modelled as MLHR, and these works have successfully adopted the Equations (26)–(31) to predict the sound absorption coefficient curves. Thus far, truss lattices of sufficiently high relative densities,^[^ ^10^, ^11^, ^13^ ^]^ plate lattices with pores,^[^ ^11^, ^17^ ^]^ and hybrid lattice structures,^[^ ^12^, ^23^, ^24^ ^]^ have been investigated. We next look at specific examples on how the Helmholtz geometrical parameters are derived from the different types of lattice structures.

Truss lattices of sufficiently high relative density (around 0.3 or more) generally present a narrow‐neck and wide‐cavity morphology. High relative density translates to thick struts. Thick struts are necessary to account for a reduced narrow neck region at the midpoints of nodes. Additionally, the large curvature of the thick struts ensures a wide cavity immediately following. Examples include the FCC truss and its derivatives. We take a look at one specific example using the fluorite crystal truss from the work of Li et al.^[^ ^11^ ^]^ A specific cut‐plane of the fluorite truss, that reveals the acoustical geometries, is shown in Figure 5C. As shown, a pore‐like region of a defined pore size, d, and depth, t, forms due to the presence of a gap between the two struts. Also, these struts need to be sufficiently close and thick such that this pore forms. For instance, trusses of low relative density does not constitute a narrow neck and wide cavity morphology.^[^ ^9^, ^10^ ^]^ Following these struts, the immediate emptiness then constitutes the cavity region of a defined size, D. It is obvious that such a truss architecture is reminiscent of a multi‐layered Helmholtz resonator.

Apart from trusses, plate lattices also constitute such a behavior. In fact, many plate lattices look physically similar to a Helmholtz resonator. We look at one FCC‐plate structure shown in Figure 5D. First brought up by Berger et al., the FCC plate structure is made up of plates that represents the slip planes of the FCC Bravais lattice.^[^ ^48^ ^]^ Since it is a closed cell structure, for 3D printing purposes, researchers introduce holes to it to remove residual materials. As can be seen, an FCC‐plate with holes look exactly like a Helmholtz resonator (Figure 5D), where the holes represent the neck, and the cell represent the cavity.^[^ ^11^ ^]^ The d, t, and effective D are marked out as shown in the same figure. Another example shown in Figure 5E is based on cubic plate enclosures, with pores artificially introduced. Alternatively, we can also regard these as Helmholtz resonators built using plates, which hence coincidentally take on the form of plate lattice structures. The previously given examples are thus far considered as simple plate lattice structures. A more complex structure, where the interconnectivity is not simply in a top‐down manner, in turn requires the further discretization of individual units and layers and the combination of series and parallel additions would be necessary.^[^ ^11^, ^84^ ^]^ For instance, the fluorite‐plate structure is shown in Figure 5F‐i.^[^ ^11^ ^]^ Discretized, it can be seen that the type of cavities are different across different layers (Figure 5F‐ii). Additionally, cavities are interconnected by the pores in parallel, apart from that in series. Therefore, two different types of layers of cavities at different layers need to be identified for the calculation of the overall acoustic impedance. The overall acoustic impedance then needs to be calculated through both series and parallel addition. Recently, there are also various hollow truss and plate lattice structures specially designed with the Helmholtz resonance mechanism in mind. Mimicking the biological features of a bamboo, Li et al. designed FCC‐based hollow trusses reinforced with plates, and with pores strategically added, to artificially introduce Helmholtz resonance chambers.^[^ ^15^, ^16^ ^]^ Volume bounded by the FCC‐arranged plates then constitute the cavity region. The work also reveals that these specially introduced resonant mechanisms enable a significantly more effective sound absorption than that of the original truss structures.

**Figure 6:**
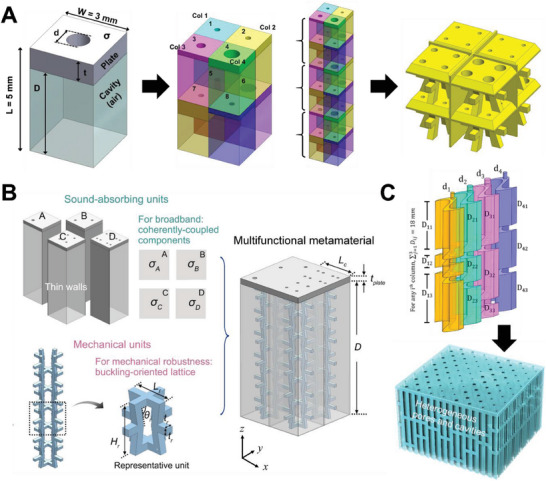
Lattices based on MLHR with heterogeneous geometries. A) A schematic of the design of a heterogeneous lattice absorber, revealing a single unit cell, a macro‐cell consisting of an assembly of optimized geometries, and an illustration of the final heterogeneously structured lattice design. Reproduced with permission.^[^ ^12^ ^]^ Copyright 2021, Wiley. B) A schematic of the de‐coupled design for a multifunctional lattice, involving the separate design of the sound‐absorbing units and the mechanical units. Reproduced with permission.^[^ ^24^ ^]^ Copyright 2022, Royal Society of Chemistry. C) Illustration of a cuttlebone‐inspired lattice absorber and its heterogeneously structured individual cells. Reproduced with permission.^[^ ^23^ ^]^ Copyright 2023, Wiley.

Finally, there are also lattices specifically designed to mimic the structure of MPPs. In general, these lattices are composed of alternating layers of plates that are embedded with perforations and lattice features, resembling a layer‐by‐layer structure. Li et al. proposed a new type of anisotropic lattice consisting of alternating layers of plates and auxetic struts.^[^ ^12^ ^]^ The plate layer consists of pores while the strut layer consists of struts that supports the plates together (**Figure** **6**A). With a “simpler” acoustical structure as compared to the previously mentioned lattice structures (for instance, in Figure 5C–F), parameters d, t and D are easily observed or defined. Also, leveraging these increased degrees of freedoms in the design (as opposed to cubic lattices), heterogeneous cells can be introduced easily. As mentioned in Section 6.2.2, heterogeneity improves the absorption bandwidth. The authors hence adopted Equations (26)–(31) for the optimization of the set of d, t, D that gives the highest effective bandwidth (Figure 6A). With eight cells in one macro‐cell, significantly improved broadband absorption has been achieved, as compared to uniform cells. The similar concept has also been adopted for the design of lattices of lower working frequency range.^[^ ^24^ ^]^ In this work, the authors proposed a concept of “decoupled” design approach for acoustics and mechanical properties. Leveraging the mechanism of Helmholtz resonance, the sound absorption property is solely dependent on the plate (pores), while the mechanical properties are solely dependent on the struts in the cavity (Figure 6B). Shown in this work, and from previous works, features inside the cavity generally holds no significant influences to the absorption coefficient curve.^[^ ^11^, ^12^, ^24^ ^]^ The structure of a biological cuttlebone is characterized by wavy cell walls bounded by plates in a layer‐by‐layer arrangement. In this regard, Li et al. utilized this structure to develop a heterogeneously porous cuttlebone‐inspired lattice absorber.^[^ ^23^ ^]^ Dissipative pores were incorporated into the plates, while partitions created by the cell walls enable the design of heterogeneous features (Figure 6C). The researchers conducted structural optimization of this heterogeneous architecture to achieve high absorption coefficients across a broad range of frequencies. Also, optimization on the sets of d and t were carried out to achieve the optimally broadband absorption behavior.

### 6.3 Cavity Resonance

#### 6.3.1 Effective Fluid Model

**Figure 7:**
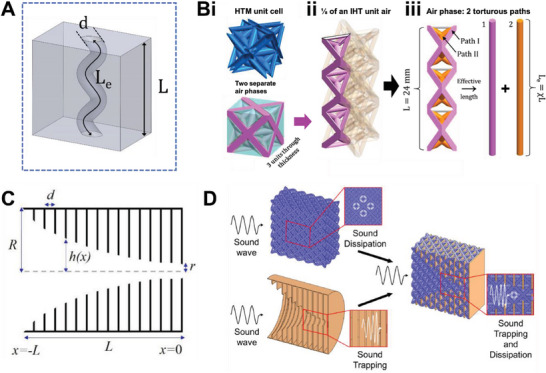
On the cavity resonance and ABH mechanisms and their related lattice structures. A) The morphology of a cavity resonator and key acoustical geometries influencing its resonant frequency. B) Schematic of the sound absorption mechanism of the hollow truss lattice, revealing i) a unit cell and the two distinct air phases, ii) a unit column of the hollow interior, and iii) discretization of the two distinct paths. Reproduced with permission.^[^ ^13^ ^]^ Copyright 2022, Wiley. C) The morphology of an SBH and key acoustical geometries influencing its resonant frequency. Reproduced with permission.^[^ ^108^ ^]^ Copyright 2022, American Institute of Physics. D) A schematic of the combination of a truss lattice, and an SBH, to form a new type of lattice structure with dual sound dissipation mechanisms. Reproduced with permission.^[^ ^9^ ^]^ Copyright 2023, Elsevier.

The cavity resonance constitutes the functioning principles of many acoustic metamaterials.^[^ ^85^, ^86^, ^87^ ^]^ Recently, it has also been employed to elucidate the mechanisms of lattice structures with hollow cavities. Thus far, one type of lattice structure reported to function based on this mechanism is the hollow truss structure.^[^ ^13^ ^]^ An example and a schematic of its principles are shown in **Figure** **7**A. In particular, geometrical parameters of interest include the strut (inner hollow part) diameter, d_s_, the unit cell thickness, L, and a derived parameter known as the tortuosity, χ. χ refers to the ratio of the actual length the sound wave travels through the structure, L_e_, to the material thickness, which is also L:^[^ ^88^ ^]^

(32) $$\begin{array}{c} \chi \;=L_{e}/L\; \end{array}$$

Physically speaking, it gives a measure of “how difficult” the sound wave propagates through the material. The key herein is therefore on deriving L_e_. Many works have proposed the division of the strut volume, by the strut cross sectional area, to obtain L_e_.^[^ ^13^, ^89^ ^]^ This way, the volume arising from the strut thickness and nodal regions are accounted for. With χ obtained, the effective fluid properties can be next derived. First, the flow resistivity is calculated:^[^ ^90^ ^]^

(33) $$\;\sigma_{r}=\frac{8\eta \chi }{\phi {\left(d_{s}/2\right)}^{2}}\;$$

ϕ refers to the porosity, per representative unit area on the surface. Where necessary, for struts inclined at an angle, ϕ can be normalized as ϕ/cos θ, where θ refers to the angle of inclination. This accounts for the increased surface porosity from the angle. The characteristic viscous length is then:^[^ ^91^ ^]^

(34) $$s\;=\sqrt{\frac{8\omega \rho_{0}\chi }{\sigma_{r}\phi }}\;$$

Following these, the effective density, ρ_e_, can then be expressed as:^[^ ^90^, ^92^ ^]^

(35) $$\;\rho_{e}=\rho_{0}\;\left(1+\frac{\sigma_{r}\phi }{i\omega \rho_{0}}G_{c}\left(s\right)\right)$$

G_c_, as a function of s, is given as:

(36) $$G_{c}\left(s\right)\;=-\frac{s}{4}\sqrt{-i}\frac{J_{1}\left(s\sqrt{-i}\right)}{J_{0}(s\sqrt{-i)}}/\left[1-\frac{2}{s\sqrt{-i}}\frac{J_{1}\left(s\sqrt{-i}\right)}{J_{0}(s\sqrt{-i)}}\right]\;$$

J_0_ and J_1_ refer to the zeroth and first order Bessel functions, respectively. The effective bulk modulus, K_e_, is in turn:^[^ ^90^, ^92^ ^]^

(37) $$\;K_{e}=\frac{\gamma P_{0}}{\left[\gamma -\left(\gamma -1\right)F\left(B^{2}\omega \right)\right]}\;$$

F, as a function of B^2^ω, is given as:

(38) $$F\;\left(B^{2}\omega \right)=1/\left[1+\frac{\sigma_{r}\phi }{iB^{2}\omega \rho_{0}\chi }G_{c}\left(Bs\right)\right]\;$$

*B* ^2^ refers to the Prandtl number as given by $\eta /(\rho_{0}\upsilon^{\prime })$ and $\upsilon^{\prime }=\kappa /\left(\rho_{0}c_{p}\right)$. The acoustic impedance, *Z*_H0_, and the effective wavenumber, *k*_e_, through the hollow cavity is thus:

(39) $$\;Z_{H0}=\sqrt{K_{e}\rho_{e}}\;$$

(40) $$\;k_{e}=\;\omega \sqrt{\frac{\rho_{e}}{K_{e}}}$$

Following these, the specific acoustic impedance of the hollow cavity, Z_H_, of the lattice structure is then given as:^[^ ^90^ ^]^

(41) $$Z_{H}=\;-i\frac{Z_{H0}}{\phi }\cot\left(k_{e}L_{e}\right)$$

With Z_H_, the absorption coefficients of the hollow structure can then be calculated using Equations (14) and (15).

#### 6.3.2 Examples

The work of Li et al. demonstrated that hollow truss lattice structures can harness the cavity resonance dissipation mechanism.^[^ ^13^ ^]^ The structure of this hollow truss is shown in Figure 7B‐i. Can be seen, there are two air phases, fully partitioned by the hollow shell, associated with the structure. There are hence two underlying mechanisms–the MLHR and cavity resonances, for sound energy dissipation. Despite its complex architecture, the structure can nonetheless be discretized into smallest units (Figure 7B‐ii). The inner hollow truss part constitutes the cavity resonance. As observed, two distinct complete tubular paths, characterized by their angle of inclination, can be proposed (Figure 7B‐iii). Given the strut diameter, d_s_, and the struts’ volume, χ and the derivatives of χ can be derived. Calculations for the overall impedance of the two paths can then be calculated using methods as of Equations (32)–(41). In turn, the multi‐layered Helmholtz resonance part of the outer truss is calculated as of methods described in Section 6.2.1. The overall impedance of the hollow truss is then calculated through summation in parallel, using Equation (31).

### 6.4 Effective Property Models

For lattice structures without obvious resonance mechanisms, e.g., pore‐and‐cavity or cavity morphologies, or lattice structures with pore sizes within the length scale of the viscous boundary layer, their acoustic impedance can in turn be derived using empirical/phenomenological methods through their effective properties. Popular mathematical models used for modelling include the Delany‐Bazley (DB) model^[^ ^93^ ^]^ and the Johnson‐Champoux‐Allard (JCA) model.^[^ ^94^, ^95^ ^]^

#### 6.4.1 Delany‐Bazley Model

The DB model is one of the first empirical models proposed for determining the bulk acoustic properties of porous materials.^[^ ^93^ ^]^ Empirically‐derived based on a large number of measurements on fibrous materials with porosities close to 1, this model calculates the characteristic specific acoustic impedance, Z_c_, and wave number, k_c_, of the material according to the following expressions:

(42) $$\;Z_{c}=\rho_{0}\;c_{0}\left[1+9.08{\left(10^{3}\frac{f}{\sigma_{r}}\right)}^{-0.75}-j11.9{\left(10^{3}\frac{f}{\sigma_{r}}\right)}^{-0.73}\right]$$

(43) $$\;k_{c}=\frac{\omega }{c_{0}}\;\left[1+10.8{\left(10^{3}\frac{f}{\sigma_{r}}\right)}^{-0.70}-j10.3{\left(10^{3}\frac{f}{\sigma_{r}}\right)}^{-0.59}\right]$$

As can be seen, the key factor influencing *Z*_c_ and *k*_c_ is σ_r_, the airflow resistivity of the material. Therefore, the acoustic impedance of such porous materials is sufficiently described solely by *σ*_r_. The flow resistivity can either be experimentally measured or numerically determined. To derive *σ*_r_ numerically we first need to work out the tortuosity, χ, and to consider the cell size, *d*_r_, of a representative volume element (RVE). A general method to derive tortuosity has been described in Section 6.3.1. For such types of porous media, *σ*_r_ is related to *χ* via the following relationship:

(44) $$\;\sigma_{r}=\frac{36\eta \chi \left(\chi -1\right)}{\phi^{2}d_{r}^{2}}$$

The range of validity of the above model is usually taken to be $0.01<\frac{f}{\sigma_{r}}<1$ and it works best when the porosity of the material is close to 1.^[^ ^95^, ^96^ ^]^ Variations of the DB model exists, for instance, alternative forms of Equations (42) and (43) as reported by Allard and Champoux are as follows:^[^ ^97^ ^]^

(45) $$\;Z_{c}=\rho_{0}\;c_{0}\left[1+0.057{\left(\frac{\rho_{0}f}{\sigma_{r}}\right)}^{-0.754}-j0.087{\left(\frac{\rho_{0}f}{\sigma_{r}}\right)}^{-0.732}\right]$$

(46) $$\;k_{c}=\frac{\omega }{c_{0}}\;\left[1+0.0978{\left(\frac{\rho_{0}f}{\sigma_{r}}\right)}^{-0.700}-j0.189{\left(\frac{\rho_{0}f}{\sigma_{r}}\right)}^{-0.595}\right]$$

#### 6.4.2 Johnson‐Champoux‐Allard Model

As compared to the DB model, the JCA model is in turn a semi‐phenomenological model that includes the viscous and thermal dissipative effects inside the porous media. Thus, this model takes into consideration dissipation at the viscous and thermal boundary layers, where the density ($\tilde{\rho }$) and bulk modulus ($\tilde{K}$) of air are in turn now based on complex functions. Their initial expressions as proposed by Johnson et al. are as follows:^[^ ^91^, ^98^ ^]^

(47) $$\;\tilde{\rho }=\frac{\rho_{0}}{\phi }\;\left[\alpha_{\infty }+\frac{\sigma \phi }{j\omega \rho_{0}}\sqrt{1+j\frac{4\alpha_{\infty }^{2}\eta \rho_{0}\omega }{\sigma^{2}\Lambda^{2}\phi^{2}}}\right]$$

(48) $$\;\tilde{K}=\frac{\gamma P_{0}/\phi }{\gamma -\left(\gamma -1\right){\left[1-j\frac{8\kappa }{{\Lambda^{\prime }}^{2}C_{p}\rho_{0}\omega }\sqrt{1+j\frac{{\Lambda^{\prime }}^{2}C_{p}\rho_{0}\omega }{16\kappa }}\right]}^{-1}}\;$$

The key geometrical parameters influencing the acoustical properties in the JCA model also include σ_r_, ϕ, χ, but additionally, the viscous characteristic length (Λ) and thermal characteristic length Λ′. Λ and Λ′ are related to the reticulation rate, R_w_, which is a measure of the area proportion of pores that are open to airflow. Modifications and variations to the JCA model for improved accuracies have also been proposed. For instance, the complex bulk modulus $\tilde{K}$ was modified by Lafarge et al. in order to better account for the thermal effects at the low frequency, thereby constituting the Johnson‐Champoux‐Allard‐Lafarge (JCAL) model.^[^ ^99^ ^]^ Later on, the JCAL model was further refined by Pride et al.^[^ ^100^ ^]^ to account for pores with possible constrictions between them, constituting the Johnson‐Champoux‐Allard‐Pride‐Lafarge model.

Here, the characteristic specific acoustic impedance (*Z*_c_) and wavenumber (*k*_c_) of a porous medium calculated using the JCA model is:

(49) $$\;Z_{c}=\sqrt{\tilde{\rho }\tilde{K}}\;$$

(50) $$\;k_{c}=\;\omega \sqrt{\frac{\tilde{\rho }}{\tilde{K}}}$$

The specific impedance through a porous medium of thickness L, modelled using either effective parameter models, is then:

(51) $$\;Z_{E}=\;-\frac{jZ_{c}\cot\left(k_{c}L\right)}{\phi }$$

#### 6.4.3 Examples

Thus far, the DB model has been used to model the absorption coefficients of truss lattice structures of very high porosities (low relative densities). As discussed in Section 6.2.3, truss lattices of high relative densities are better modelled as MLHR. In the work of Lai et al., the DB model has successfully been used to predict the sound absorption coefficients of SC trusses below relative densities of 0.3.^[^ ^10^ ^]^ Discretizing an RVE of the SC truss, χ and d_r_ of the lattice are both modelled after relationships proposed by Fourie et al. on a similar type of structure.^[^ ^88^ ^]^ σ_r_ is then calculated accordingly using Equation (44). Boulvert et al. in turn adopted the JCAL model for the design of a functionally graded lattice structure.^[^ ^25^ ^]^ The authors demonstrated that the six parameters of the JCAL model can be derived through FEM using the RVE. After deriving the structural‐property relationships, the JCAL parameters were used to optimize the absorption bandwidth through the implementation of graded layers. Nonetheless, these effective property models are more often than not used for modelling the properties of foams. Interestingly, upon discretized into RVEs, the microstructures of these foams are often proposed to be either cubic or tetrakaidecahedron truss unit cells.^[^ ^89^, ^101^ ^]^ These RVEs generally have cell sizes below 500 µm, on the order of the length scale of the viscous boundary layer thickness as governed by Equation (1). Therefore, foams can be regarded as truss lattices of sub‐millimeter unit cells. Important DB or JCA model parameters are calculated through microstructural analysis of the RVEs. Thus, if the lattices are to be manufactured at this length scale, DB and JCA models would be more effective than resonance‐based models for modelling acoustics properties.

### 6.5 Slow‐Sound Effect

#### 6.5.1 Sonic Black Hole Model

A recently discovered approach to sound absorption comes in the form of the sonic black hole (SBH). SBH derives from their structural counterpart–the acoustic black hole (ABH). The term ABH, initially introduced by Mironov,^[^ ^102^ ^]^ pertains to the investigation of flexural wave propagation within a beam that gradually tapers to zero thickness over a finite interval. This study revealed that as the thickness diminishes, the velocity of flexural waves also decreases, causing the incoming wave to gradually decelerate and preventing it from reaching the tip with a complete termination. Comparatively, SBH is proposed as the counterpart of structural ABH to absorb sound waves in air.^[^ ^103^ ^]^ It typically consists of a duct structure with decaying inner radii, where a set of rigid rings are added to provide a smoothly varying impedance boundary. The damping and viscous dissipation in such a structure is significantly enhanced due to the slowing down of sound waves and energy concentration.^[^ ^104^ ^]^ Recently, acousticians have identified that the source of acoustic energy attenuation is highly associated with resonance in both along the length of the structure as well as within the lateral cavities.^[^ ^105^, ^106^, ^107^ ^]^

Involving a circular duct with annular rings of decreasing inner radius, the SBH represents a unique sound manipulation concept where incoming sound waves are directed. Figure 7C displays an illustration of the SBH duct alongside its significant design parameters.^[^ ^108^ ^]^ Based on the pioneering work by Mironov,^[^ ^103^ ^]^ the governing equation of sound propagation in the SBH structure is as follows:

(52) $$\frac{\partial^{2}p}{\partial x^{2}}+\frac{\partial p}{\partial x}\frac{\partial }{\partial x}\left(\ln S\left(x\right)\right)+p\;\left(k_{0}^{2}+j\rho_{0}\omega \frac{2Y\left(x\right)}{h\left(x\right)}\right)=\;0$$

where p is the acoustic pressure, and h(x) and S(x) represent the radius and cross sectional area of the duct at a position along the x‐axis, respectively. The wall admittance, Y(x), as a function of position x, is represented by the following for circular ducts:^[^ ^108^ ^]^

(53) $$\begin{array}{c} Y\;\left(x\right)=\;-\frac{j\omega }{\rho_{0}c_{0}^{2}}\frac{R^{2}-h^{2}\left(x\right)}{2h\left(x\right)} \end{array}$$

The inner radius of an SBH with a quadratic decaying profile, as a function of x, can be represented as follows:

(54) $$h\;\left(x\right)=\frac{R}{L^{2}}\;x^{2}$$

*R* is the radii of the circular duct at the entrance and *L* is the length of the duct. The phase (*c*_ph_) and group (*c*_g_) velocities of sound wave propagation proposed by Mironov are as follows:^[^ ^103^ ^]^

(55) $$\;c_{ph}=\frac{\omega }{k}\;=\;-\frac{\omega }{\sqrt{k_{0}^{2}L^{2}-1}}x$$

(56) $$\;c_{g}=\;-\frac{c_{0}^{2}\sqrt{k_{0}^{2}L^{2}-1}}{L^{2}\omega }x$$

It can then be seen that $\lim_{x\to 0^{-}}c_{ph}=\;0$ and $\lim_{x\to 0^{-}}c_{g}=\;0$, which implies that both the phase and group velocities tend to zero near the termination. In this scenario, the incident sound wave is prevented from reaching the termination and instead, it is focused inside the duct without any reflection or transmission.

For a more general case where the termination radius of the duct r tends toward a finite value, the SBH profile takes the form:

(57) $$h\;\left(x\right)=\frac{R-r}{L^{2}}\;x^{2}+r$$

Based on the works by Mi et al.,^[^ ^108^ ^]^ the phase and group velocities of sound wave propagation are in turn represented as follows:

(58) $$\;c_{ph}=\frac{\omega }{k}\;=\frac{\omega \left[\left(R-r\right)x^{2}+rL^{2}\right]}{\sqrt{k_{0}^{2}R^{2}L^{4}-4{\left(R-r\right)}^{2}x^{2}}}\;$$

(59) $$\;c_{g}=\frac{c_{0}^{2}\left[\left(R-r\right)x^{2}+rL^{2}\right]\sqrt{k_{0}^{2}R^{2}L^{4}-4{\left(R-r\right)}^{2}x^{2}}}{\omega L^{4}R^{2}}\;$$

In turn, the phase and group velocities decrease to a finite value now, given that $\lim_{x\to 0^{-}}c_{ph}=\frac{r}{R}\;c_{0}\;$and $\lim_{x\to 0^{-}}c_{g}=\frac{r}{R}\;c_{0}$. If the R/r ratio is large, the decrease in sound speed within the SBH results in increased travelling time of sound waves that enter the structure, which thereby leads to enhanced sound absorption. Overall, the acoustical parameters that influence the sound absorption capabilities of an SBH include the length of duct (L), the SBH profile of the acoustic duct h(x), the diameter of the duct at the entrance (R), the inner diameter of the duct at the termination (r), number of rings utilised to replicate the thickness profile, thickness of each ring (t).

#### 6.5.2 Examples

A recent work demonstrated the potential of incorporating SBH into lattice structures (Figure 7D). In the work of Chua et al.,^[^ ^9^ ^]^ the authors worked on novel lattice structures based on the combination of Bravais lattice mimicking lattice structures: SC, BCC, FCC and fluorite crystals, and SBH plates. Measurements of the sound absorption coefficients of the printed samples indicated that the sound absorption coefficients of the SBH‐incorporated lattices are more than double that of the pure strut lattices, with the addition of more SBH plates increasing the overall absorption coefficients. Simulations also revealed that the incorporation of SBH plates also successfully incorporated the sound speed reduction effects of the SBH alongside the sound absorption mechanism of resonating cells inherent in truss lattice structures. The SBH plates have the effects of focusing most of the sound power dissipation within the SBH cavity while decreasing the sound speed and frequency of the sound waves. By positioning the SBH plates to coincide with the locations of the necks of the resonant cells in the truss lattices, the energy dissipation density at the resonant cells can be amplified.

## 7 Finite Element Analysis

**Figure 8:**
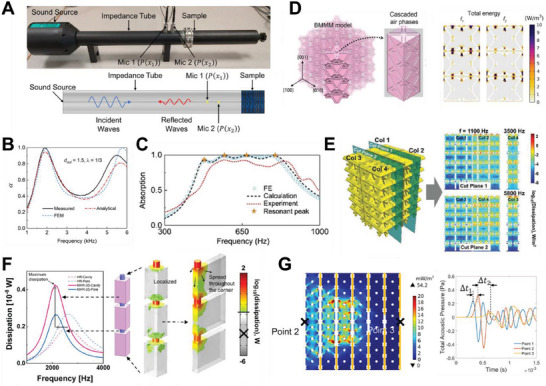
Modelling sound absorption in lattice structures using FEA. A) An illustration of the actual impedance tube and the FEA model, with relevant features annotated. Reproduced with permission.^[^ ^9^ ^]^ Copyright 2023, Elsevier. B,C) show the correlation of the experimentally measured and FEA modelled sound absorption coefficient curves. Reproduced with permission.^[^ ^14^ ^]^ Copyright 2023, American Chemical Society. Reproduced with permission.^[^ ^24^ ^]^ Copyright 2022, Royal Society of Chemistry. D,E) show the energy dissipation through the lattice structures, shown on selected cross sectional planes. Reproduced with permission.^[^ ^16^ ^]^ Copyright 2023, American Chemical Society. Reproduced with permission.^[^ ^12^ ^]^ Copyright 2021, Wiley. F) Illustrations of the energy dissipation in an MLHR and a modified MLHR, revealing the extended depth of dissipation in modified MLHRs. Reproduced with permission.^[^ ^82^ ^]^ Copyright 2023, Royal Society of Chemistry. G) Energy dissipation and the transient acoustic pressure observed through SBH lattices. Reproduced with permission.^[^ ^9^ ^]^ Copyright 2023, Elsevier.

Numerical simulation, in terms of finite element analysis (FEA), has also been widely employed for the prediction of sound absorption performances in lattice structures. There are two main purposes for FEA herein, first, to predict absorption, and second, to observe locations with the highest sound energy losses. Different from analytical analysis, in FEA, discretization of the geometry is not needed. In turn, the entire model is implemented for simulation. For modelling sound absorption in lattice structures, FEA methods adopt the physics of thermal and viscous boundary losses. FEA is mainly carried out using COMSOL Multiphysics, using the acoustics modules.^[^ ^9^, ^12^, ^14^, ^15^, ^16^, ^24^, ^82^ ^]^ Typically, simulation is conducted by modeling the actual impedance tube using the transfer function specified in accordance with ASTM E1050‐19 standards. An example is shown in **Figure** **8**A.^[^ ^9^ ^]^ As opposed to the actual physical model, FEA is carried out instead using the air phase with the appropriate boundary conditions applied. For this example, and most of the cases, the Pressure Acoustics and Thermoviscous Acoustics modules in COMSOL Multiphysics have been applied to the impedance tube and sample, respectively. The purpose of the pressure acoustics module is for the generation of incident sound waves and the collection of pressure data points. To mimic the actual tube, which is made of thick solid steel or polymer, boundaries are introduced such that no outward travel of sound waves is possible. The lattice structure is in turn modelled using Thermoviscous Acoustics for detailed viscous and thermal boundary losses upon wave interactions. The principle of loss in the thermoviscous acoustics module in COMSOL is based on the viscous and thermal boundary layers under the assumption of isentropic acoustics and a lossless system. Boundaries of the samples are in turn modelled such that the airflow will have zero velocity relative to the (hypothetical) walls of the sample. The transformation and equations for the calculation of the sound absorption coefficients are then calculated in accordance with Equations (5)–(11), starting with the calculation of sound pressures (P(x_1_) and P(x_2_)) at the specified integration points on the FEA model. The absorption coefficient curve is then obtained by calculations across the entire range of frequencies. Dissipation information is then extracted via the in‐built physics of the Thermoviscous Acoustics module.

Figure 8B,C depicts the correlation between experimental data and the FEA modelled absorption curves, demonstrating the high‐fidelity modeling capabilities using finite element models.^[^ ^14^, ^24^ ^]^ Figure 8D,E then reveals some of the FEA modelled sound dissipation distribution in MLHR‐based lattice structures.^[^ ^12^, ^16^ ^]^ In line with the Helmholtz resonance principle, the highest dissipation is always observed at the pores, or necks, or reduced regions of the lattice. In the work of Li et al., FEA has been dedicated for the purpose of investigating the unprecedented enhancement in sound dissipation observed in MLHR of modified geometries.^[^ ^82^ ^]^ For example, FEA dissipation plots enable us to observe how thermal and viscous dissipations extend and become enhanced when the pore of the MLHR is brought into close proximity to its cavity wall (Figure 8F). Shown in Figure 8G, FEA has also been purposed for illustrating the energy dissipation through SBH lattices, and plotting out the respective transient acoustic pressure loss.^[^ ^9^ ^]^ Overall, FEA complements the modeling of absorption coefficient curves and provides a comprehensive illustration of sound dissipation within the structure.

## 8 Structure‐Mechanism‐Property Relationship

Given the current research progress on sound‐absorbing lattice structures, we have classified four primary sound absorption mechanisms related to lattice structures. Considering the various types of lattice structures, we aim to propose a collective structure‐mechanism‐property relationship based on lattice features, mechanisms, and sound absorption properties. Herein, in terms of acoustically active geometry, we classify the lattices into three classes: (i) lattices with small pores and large cavities, (ii) lattice with uniform and continuous cavities, and (iii) lattices with uniform pores and cavities.

### 8.1 Lattice Classification According to Acoustics

#### 8.1.1 Lattices with Small Pores and Large Cavities

**Figure 9:**
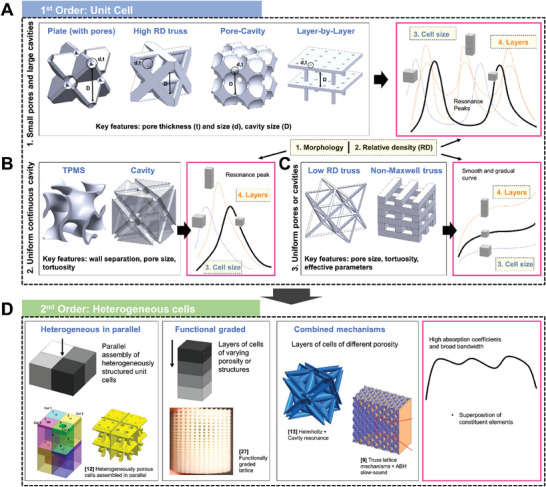
An overview of the structural‐mechanism‐property relationship of lattice structures for sound absorption, and their classification at the unit cell level and beyond the unit cell level. At the unit cell level, A) lattices with small pores and large cavities, B) lattices with uniform and continuous cavity, and C) lattices with uniform pores or cavities. The key acoustical geometries that influence the sound absorption coefficient curve, and the influence of various lattice parameters, are included. As shown, different classes of cells result in different types of sound absorption curves. D) Classification of lattices beyond the unit cell level. Beyond the unit cell, lattices can be combined in parallel, functional graded, or with several distinct sound absorption mechanisms. In this case, the sound absorption curve is usually a superposition of its constituent elements of expanded absorption bandwidth. Reproduced with permission.^[^ ^12^ ^]^ Copyright 2021, Wiley. Reproduced with permission.^[^ ^27^ ^]^ Copyright 2019, Elsevier. Reproduced with permission.^[^ ^13^ ^]^ Copyright 2022, Wiley. Reproduced with permission.^[^ ^9^ ^]^ Copyright 2023, Elsevier.

These lattices form the bulk of the lattices that are acoustically active. These lattices include dense trusses, plate lattices with pores, lattices with elaborately designed pores and cavities. Illustrations of their common structures are shown in **Figure** **9**A. Although drastically different appearance wise, one common feature they share is that they all display a narrow pore and large cavity structure. Figure 9A thus also illustrates the identification of the pore and wide cavity in these different structures. For dense trusses, pores are observed at the intersection of struts while anything below constitutes the large cavity. For plate lattices with pores, and lattices with elaborately designed pores and cavities, this identification is rather straightforward. Sometimes, they are in turn designed with this consideration in mind. For this morphology, their sound absorption mechanism is attributed to Helmholtz resonance and their absorption curves are characterized by resonance peaks with high maximum and narrow bandwidth. For these structures, the surface porosity of the pore should be ideally as low as possible, for instance, ratios are commonly less than 10%. Large ratios result in the deviation away from Helmholtz resonance. The resonant frequency of absorption is in turn solely dependent on the acoustical geometrical features and can be calculated in accordance with Equations (25)–(30). This also implies that any other features of the lattice, apart from its critical acoustical features, have no influences on its sound absorption properties. Of course, if the features were to interfere with the acoustical features, such as the D spacing as described in Section 6.2.1, slight modifications to the variable would be necessary in order to obtain an accurate prediction.^[^ ^11^, ^24^ ^]^ Therefore, despite having drastically different structures (Figure 9A), these lattices all display the similar type of sound absorption coefficient curves.

#### 8.1.2 Lattices with Uniform and Continuous Cavities

Lattices that fall under this category include various types of sheet TPMS structures and lattices based on the inverse of trusses.^[^ ^13^, ^21^ ^]^ As schematically illustrated in Figure 9B, these lattices are associated with highly uniform and continuous air cavities. For instance, owing to their zero mean curvature design, the sheet gyroid consist of continuous sheets of a constant separation distance.^[^ ^21^ ^]^ They thus do not display a narrow neck and wide cavity morphology and hence are unable to display the Helmholtz resonance behavior. Sound absorption in these lattices is based on the viscous and thermal dissipations through structural resonance, very much similar to that of acoustic metamaterials.^[^ ^86^ ^]^ Being in a state of resonance, these lattices display absorption peaks, like that of Helmholtz resonators (Figure 9B).

#### 8.1.3 Lattices with Uniform Pores and Cavities

Finally, another class is lattices with only uniform pores and cavities. Such lattice structures usually consist of low relative density trusses,^[^ ^9^, ^10^ ^]^ woodpile trusses,^[^ ^26^ ^]^ functionally graded truss.^[^ ^25^ ^]^ As schematically illustrated in Figure 9C, these lattices have comparatively much more uniform pores and cavities as compared to those mentioned in Section 8.1.1. For instance, low relative density trusses do not display a reduced neck region owing to their thin struts, and woodpile trusses do not have nodes that will construct a reduced neck region. Similarly, these lattices do not experience the intense dissipation during Helmholtz resonance, and they can only harness viscous and thermal dissipation based on the resistance to the airflow. Absorption properties of these lattices can better be predicted using effective property models. Their properties are essentially foam‐like and they thus display smooth and gradual absorption curves (Figure 9C).

### 8.2 Heterogeneous Features

The previous section provided an overview on the types of lattices in terms of acoustics. Thus far, researchers have also focused on developing heterogeneous lattice structures that combine various acoustic mechanisms and hence physical features to achieve enhanced sound absorption performance, both in terms of absorption and bandwidth. Schematically illustrated in Figure 9D, the most commonly studied heterogeneous lattice are those based on heterogeneous cells in parallel,^[^ ^12^, ^23^, ^24^ ^]^ functionally graded cells,^[^ ^25^, ^26^, ^27^, ^28^ ^]^ and lattices with a combination of mechanisms.^[^ ^9^, ^13^ ^]^ For the heterogeneous cells in parallel, majority take on the form of a macro‐cell comprising of Helmholtz resonators with different resonant frequencies. In this case, numerous unique dissipation media exist, and multiple resonance modes thus work in tandem, resulting in an overall absorption curve superimposed from these constituents. Broadband absorption can thus be effectively achieved in Helmholtz resonators this way. Similar to heterogeneous lattice in parallel, functionally graded lattices harness the superposition of different absorption regions arranged in series. These lattices are usually based on unit cells with increasing, decreasing, or varying relative densities along sound incidence. Thus far, lattices consisting of heterogeneous mechanisms include hollow trusses and trusses with SBH. For hollow trusses, heterogeneity is achieved here through subtraction, via removing the inner materials of the truss. Thus, two dissipation media, one based on the truss exterior, and another on the truss hollow interior, are obtained.^[^ ^13^ ^]^ As revealed in the study, a desirable combination of exterior and interior geometry enables the optimal gain in absorption.^[^ ^13^ ^]^ Also, truss lattices with SBH incorporated have been investigated.^[^ ^9^ ^]^ As demonstrated in this study, absorption is dominated by the SBH, which significantly enhanced the absorption properties of the lattice structures.

### 8.3 Influence of Lattices Parameters

Lattices structures are characterized and defined by several features. Intrinsic properties defining lattices include their (i) morphology and (ii) relative density. Considering the same material, the mechanical properties of lattices are solely dependent on these two intrinsic properties. However, differing from mechanical properties, acoustical properties are also highly dependent on the extrinsic factors, namely the (iii) cell size and (iv) number of cells along the sound propagation direction. Factors (i)–(iii) have a direct impact on the pore and cavity morphologies that sound waves propagate through in the lattice structure, while factor (iv) influences the overall depth of sound wave dissipation in the medium. In this section, we aim to provide an overview of the influence of these factors on the sound absorption coefficients of lattice structures, based on the current literature.

#### 8.3.1 Morphology

The morphology, e.g. whether the lattice consists of struts, plates, shells, or any other features, directly influences the lattice features (e.g., pore size, strut size, cell wall separation, cavity size, etc.) and hence the acoustical geometries. However, there is no direct formula or rule that relates the lattice features to the sound absorption coefficients of the lattice. Absorption coefficients need to be calculated through the appropriate method as summarized in Section 6. For Helmholtz resonators, the resonant frequencies depend on the combination of the set of acoustical geometries: d, t, D, and σ, as opposed to any single metric. For instance, the same resonance behavior can be achieved even for sets of drastically different geometries.^[^ ^12^, ^23^ ^]^ At any frequency (or range), optimal absorption takes place at a combination of specific geometries. Nonetheless, for Helmholtz and cavity resonators, one general rule is that the smaller the features, the more likely the effective absorption frequencies to be higher. If the lattice features are brought down to the length scale of the viscous boundary layer,^[^ ^109^ ^]^ then thermoviscous dissipation takes place via general flow resistance through a porous medium. For such lattices, the smaller the pore size, the better the intensity of sound absorption.^[^ ^101^ ^]^ For both cavity resonators and flow resistance media, in general, the higher the tortuosity, the higher the absorption.^[^ ^21^, ^89^ ^]^

#### 8.3.2 Relative Density

For lattices, the higher the relative density, the smaller the pores and cavities would be. This inadvertently influences the acoustical geometries. For Helmholtz resonance based structures, the extent of influence might be different for different parameters. For instance, the work of Li et al. reveals that for a truss lattice, dimensions d, t, and D are each reduced at very different rates when the relative densities increase.^[^ ^13^ ^]^ This thus implies no regular trends to the sound absorption curves in such a scenario. For cavity resonance or flow resistance based structures, a trend is slightly clearer. In general, reduced pore sizes results in the frequency range of effective absorption to increase.^[^ ^13^ ^]^ In essence, there is no direct relation between the relative density and absorption properties.

#### 8.3.3 Cell Size

There is a direct relation between the acoustical geometries of the lattice and its cell size. For the same lattice, the larger the unit cell, the lower the working frequency range. There is no correlation to the intensity of absorption, however.

#### 8.3.4 Number of Cells in the Direction of Sound Wave Propagation

Although the number of cells along sound wave propagation does not directly affect the sizes of the pores and cavities within the lattice structure, it does have a direct impact on the total depth of the dissipation medium. This thus highly influences the sound absorption properties of the lattice structure. For both Helmholtz and cavity resonators, the first resonance peak (multiple peaks for multi‐layered structures) generally shifts to the lower frequency with increasing number of layers of cells.^[^ ^11^ ^]^ This attributes to the longer air cavities (from the increased number of cells) having a better impedance match and hence stronger acoustical interactions with waves that are periodically longer (lower frequency). For Helmholtz resonators, the number of peaks increases with increasing number of cell layers.^[^ ^11^ ^]^ However, there is no direct relation between the number of layers and the intensity of absorption. Absorption maximum and bandwidth may or may not broaden with more layers. For Helmholtz resonators, it has been observed that with increasing number of layers, certain geometries can lead to a decrease in resonance peaks, widening of the bandwidth, and a flattened absorption curve.^[^ ^11^ ^]^ For flow resistance based structures, the intensity of absorption increases with increasing thickness.

## 9 Potential for Multifunctionality

Lattice structures are initially designated as lightweight materials and energy absorbers for various applications in aerospace, automotive, construction, and sports, etc. Intuitively, the reduction of materials renders them to be lightweight. Then, being porous, the unique geometric arrangement of lattices allows them to absorb energy in a controlled manner, reducing the mechanical force experienced by the target of protection. Therefore, in addition to their sound absorption capabilities, these materials inherently serve the aforementioned purposes, making them multifunctional materials.

**Figure 10:**
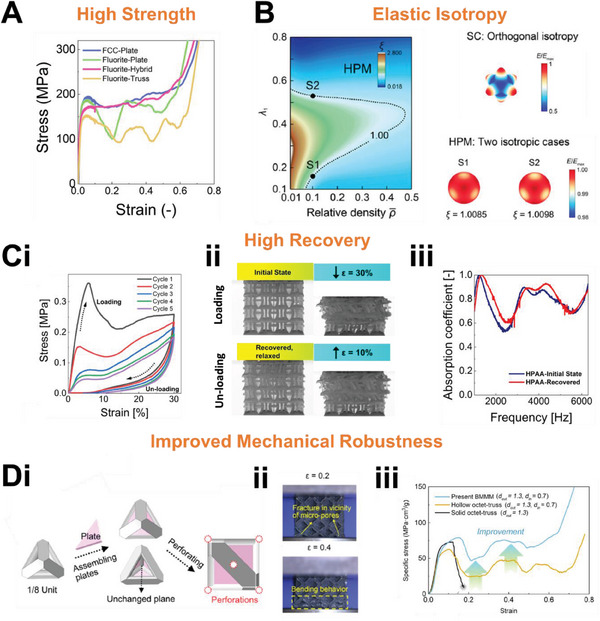
An overview of the mechanical properties of lattice sound absorbers. A) Metallic lattices with high strength and SEA. Reproduced with permission.^[^ ^11^ ^]^ Copyright 2021, Wiley. B) Sound absorbing lattice design also incorporating elastic isotropy structural optimization. Reproduced with permission.^[^ ^17^ ^]^ Copyright 2022, Taylor & Francis. C) Polymeric auxetic lattice with high strain recovery, revealing i) cyclic compression stress‐strain curves with high strain recovery. ii) Digital images of the compressive deformation, revealing recovery, and iii) the sound absorption curves before and after compression‐recovery. Reproduced with permission.^[^ ^12^ ^]^ Copyright 2021, Wiley. D) Simultaneously enhanced mechanical robustness from the introduction of sound dissipation mechanisms. D‐i) Schematic of the bamboo‐inspired lattice design, D‐ii) its compression stress‐strain curve and as compared to its constituent designs, and D‐iii) captures of the deformation sequence during compression, revealing deformation tolerance. Reproduced with permission.^[^ ^16^ ^]^ Copyright 2023, American Chemical Society.

Several works have shown the potential of lattice structures for both functions. Thus far, these lattices have been fabricated using various materials, ranging from high strength metals to tough polymers. Lattices fabricated using metals have shown the potential as ultrastrong, temperature resistant lightweight materials for use under harsh environments.^[^ ^11^, ^16^, ^17^ ^]^ For instance, plate lattices made using steel has shown to display a high strength up to 205 MPa at a low density of 2.4 g cm^−3^ (**Figure** **10**A). It also displays unprecedented toughness with a specific energy absorption (SEA) of 50.3 J g^−1^. Properties derive from both the plate morphology and the strong and ductile base material used. Therefore, these lattices can serve as alternatives to aluminium due to their similar properties, and in addition, they can also function as sound and energy absorbers. Authors have also demonstrated the potential of incorporating structural optimization to achieve elastic isotropy in sound‐absorbing lattices (Figure 10B).^[^ ^17^ ^]^ Such lattices thereby constitute lightweight structures consistent in multiple directions. Using a tough polymer, sound‐absorbing lattices which are also deformation recoverable have been demonstrated.^[^ ^12^, ^24^ ^]^ For instance, one lattice displays simultaneously high strain recovery (up to 30%) and strength (0.37 MPa) as depicted in the cyclic compression tests (Figure 10C‐i).^[^ ^12^ ^]^ The heterogeneous Helmholtz resonance cell assembly is responsible for the broadband sound absorption, whereas the deformation recovery is attributed to both the inherent toughness of the polymer and the intentionally‐incorporated auxetic struts (Figure 10C‐ii). Such a sound absorber can be considered pseudo‐reusable, as it exhibits minimal changes in sound absorption after impact and recovery (Figure 10C‐iii).

Sometimes, the deliberate introduction of sound dissipating cells/features also promotes better mechanical robustness. For instance, through the deliberate introduction of Helmholtz resonance compartments to FCC trusses, authors have developed a new class of bamboo‐inspired tube‐plate hybrid lattice that exhibit improved SEA (Figure 10D‐i).^[^ ^15^, ^16^ ^]^ The addition of plates reveals both improved toughness and strength from its truss and tube constituents, respectively (Figure 10D‐ii,iii). Leveraging heterogeneous cavity depths (D) to achieve broadband absorption in a multi‐cell lattice, another work revealed the potentials of enhanced deformation tolerance in such a structure as compared to a layer‐by‐layered one.^[^ ^23^ ^]^ This is akin to the biomimicry of naturally occurring staggered biological structures. Hollow truss lattices, with dual sound dissipation mechanisms, in turn display elongated stress plateaus as compared to their solid truss counterparts.^[^ ^13^ ^]^ This attributes to the transition from the highly stretching‐dominated behavior of the solid truss to the bending‐dominated behavior of the hollow truss. Considering features, truss lattices with SBH introduced also reveal enhanced SEA and absorption efficiency, primarily owing to the SBH plates aiding in mitigating abrupt fracture and stress valleys.^[^ ^9^ ^]^

## 10 Future Outlook

### 10.1 Recommendations for Design

The structural property relationships of various types of lattices have been reviewed in Section 8. It is clear that effective sound absorption properties (e.g, α ≥ 0.7) are achieved only at optimal sets of geometries. Therefore, as opposed to studying any conventional lattice design, we recommend proper design and optimization based on mechanisms. A brief guide is outlined in this section. Thus far, the Helmholtz resonance mechanism has been revealed to be among the most useful routes for sound absorption. This mechanism is most adopted in truss, plate, or layer‐by‐layer plate lattices. The condition for Helmholtz resonance includes a narrow pore (neck) followed by a larger cavity. For this morphology to be present, for trusses, the lattices need to be either sufficiently dense (e.g., relative density of > 30%), or to be sufficiently complex (e.g., high Maxwell's number). In turn, plate lattices achieve this morphology with simply pores introduced. It is also important to design optimal geometries for cavity resonators. For cavity resonators, such as tubes or TPMS, it is to note that the higher the tortuosity, the better the absorption.^[^ ^21^ ^]^ Therefore, one should leverage structural complexity as a consideration herein. If no resonance mechanisms are to be present, structures can be designed or scaled to have pore sizes at the length‐scale of the viscous boundary layer. Lattices at this length‐scale display absorption coefficient curves reminiscent of foams, which is gradual and smooth. While homogeneous lattices can exhibit characteristic resonance peaks near maximum absorption, the introduction of heterogeneity is strongly recommended to achieve an ultra‐wide bandwidth of absorption. This can be achieved by either parallel or series arranged assembly of cells with different structural features. Further, heterogeneity in mechanisms can be considered, e.g. as demonstrated, introducing cavity resonance in hollow trusses,^[^ ^13^ ^]^ and introducing slow‐sound effect with SBHs.^[^ ^9^ ^]^

### 10.2 New Concepts

**Figure 11:**
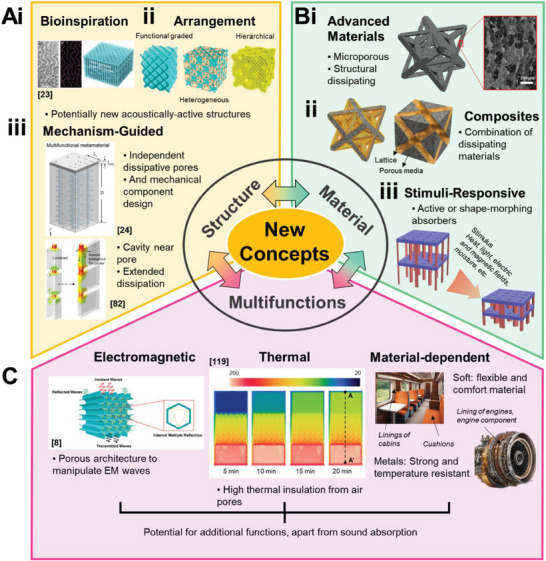
An overview of the new concepts possible for sound‐absorbing lattice structures. A) New concepts in terms of the structure, including i) bioinspiration, ii) non‐uniform arrangements of unit cells, and iii) acoustics mechanism‐guided design. Reproduced with permission.^[^ ^23^ ^]^ Copyright 2023, Wiley. Reproduced with permission.^[^ ^24^ ^]^ Copyright 2022, Royal Society of Chemistry. Reproduced with permission.^[^ ^82^ ^]^ Copyright 2023, Royal Society of Chemistry. B) New concepts in terms of material, including i) adopting advanced materials as the base material, ii) creating lattice and dissipative phase composites, and iii) using stimuli‐responsive materials to create active or morphing absorbers. C) Potential new functions, apart from sound absorption, including electromagnetic, thermal, and material‐dependent meta‐properties. Reproduced with permission.^[^ ^8^ ^]^ Copyright 2022, Royal Society of Chemistry. Reproduced with permission.^[^ ^119^ ^]^ Copyright 2021, Elsevier.

Sound‐absorbing lattice structures have immense potential for innovation and development. Thus far, research carried out for sound‐absorbing lattice structures is minuscule of all the possible potentials. In terms of new concepts, researchers can consider the following factors, but not limited to: (i) structural design and concept, (ii) materials selection, and (iii) functionalities. Some of the advanced concepts adopted for the design of mechanically robust lattices can also be applied for the design of sound‐absorbing lattices. These include unit cell design such as bioinspiration, inspiration based on naturally occurring matter (**Figure** **11**A‐i), and macro‐level design such as heterogeneous, fractal, hierarchical, and graded structures (Figure 11A‐ii). Thus far, a bioinspired cuttlebone lattice with heterogeneous cells have displayed the potential to achieve simultaneous broadband absorption and mechanical robustness. Various works also demonstrated the potential of graded lattices. Nonetheless, we foresee further potentials considering the vast of collection of biological structures and the untapped design concepts. Also, the exploration of new structural designs may lead to the discovery of improved or even entirely new dissipation mechanisms, beyond those discussed in Section 6. For instance, improved sound dissipation in MLHR lattices can be achieved when the pores are positioned near the cavity, demonstrating the potential for enhanced dissipation.^[^ ^82^ ^]^ This highlights the potentials to achieve unprecedented sound dissipation properties in newly developed structures, and also on the potentials to design new lattice structures based on leveraging dissipation mechanisms (Figure 11A‐iii). As the primary dissipation in a Helmholtz resonator predominantly occurs at the neck, Li et al. demonstrated that the sound absorption characteristics of a MLHR can be independently designed by focusing on the neck morphology, with relatively little consideration given to the cavity.^[^ ^24^ ^]^ This implies that the absorption properties and mechanical properties can be can be designed independently, opening more ways for customizable properties.

As established throughout this review, sound absorption is materials independent as long as the solid material is sufficiently rigid. This also forms the fundamental basis for the mechanisms illustrated in Section 6. However, researchers could explore the use of non‐traditional solid materials, such as advanced materials like graphene and its derivatives, as well as various types of aerogels (Figure 11B‐i), for the fabrication of lattice structures. Till date, various graphene‐derived materials and aerogels have been successfully 3D printed or templated,^[^ ^110^, ^111^ ^]^ implying the potential of fabricating lattice structures using these materials. In terms of sound absorption, these materials are composed of microporous and rough surfaces that are expected to contribute to enhanced viscous flow dissipation.^[^ ^112^ ^]^ Furthermore, their ultra‐lightweight features could lead to sound energy dissipation through vibrational damping of sound waves. Indeed, bulk aerogels have displayed tremendous potential for broadband sound absorption.^[^ ^113^ ^]^ Apart from this, composites of lattice structures could also be considered. Porous media such as foams and aerogels are highly dissipative. Researchers can also work on the lattice composites consisting of dissipative air chambers and porous media. This concept holds potentials for lattices with distinct partitioned chambers. For instance, for the hollow truss with dual air phases, one can be infilled with the porous medium while the other can continue to function as an air cavity resonator, or vice‐versa, as schematically illustrated in Figure 11B‐ii. In addition, utilizing smart materials that undergo shape changes in response to external stimuli can lead to the development of smart or active sound absorbers. By engineering structural changes in the lattice, critical acoustical parameters can be altered, resulting in different resonances or absorption intensities (Figure 11B‐iii). The potential of using light,^[^ ^114^ ^]^ heat,^[^ ^115^ ^]^ magnetic field,^[^ ^116^ ^]^ electric field,^[^ ^117^ ^]^ and moisture^[^ ^118^ ^]^ to achieve stimuli response in 3D printed components has been demonstrated. For acoustics in particular, researchers shown the potentials of magnetically‐responsive lattices with tuneable sound transmission bandgaps.^[^ ^116^ ^]^

Apart from sound absorption, other multifunctional properties are also possible considering the adoption of relevant materials and structural designs. In addition to mechanical properties, lattice structures also display potential as electromagnetic wave absorbers or thermal insulators owing to their intricate porous architecture (Figure 11C).^[^ ^7^, ^8^, ^119^ ^]^ Similarly, these properties are complementary in the intended application and the environment of use, rather than being incongruent. In terms of electromagnetic wave absorption, such materials could have potential applications in the telecommunications or defence industries, where the need for materials that can both absorb sound and electromagnetic radiation is essential. Similarly, sound‐absorbing lattices that are thermal‐insulating could prove valuable in aerospace and automobile applications, where the simultaneous generation of heat and sound energy is prevalent. Specific functionalities can also be achieved from materials selections. For instance, metals can be used when both strength and thermal resistance are necessary, as in engine applications. In contrast, elastomeric materials can be employed when human contact is required, such as for cushions or interior linings. In short, there is still much potential for further research and development in sound‐absorbing lattice structures. By exploring new design concepts, selecting new materials, and incorporating other functionalities, researchers could unlock further potentials in sound‐absorbing lattices and pave the way for new applications in a variety of industries.

### 10.3 Potential Applications

As stated in Section 9, lattice structures display the potential to multifunction as mechanical metamaterials, apart from being a sound absorber. In general, the application of lattice structures with dual capabilities of sound absorption and customizable mechanical properties, such as energy absorption, high strength‐to‐weight, are diverse and impactful across various industries, offering improved safety, energy efficiency, and comfort in a wide range of scenarios. Their adaptability to different materials and 3D printing technologies further extends their potential. Most importantly, as opposed to being incongruent, sound absorption is usually required for all these situations where energy absorption is needed, and vice versa. Some of the potential applications where these lattice structures can be implemented includes the automotive and aerospace industries. In the automotive industry, lattice structures made of soft materials could be integrated into the interior of vehicles and seats (Figure 11C). First, they serve as effective sound absorbers for noise control. Then, in the event of a collision or sudden deceleration, they would be able to absorb and dissipate kinetic energy, reducing the risk of injury to occupants. This not only enhances passenger safety, but also contributes to effective material usage. Lattices made of strong and rigid metals can in turn be used as critical structural components, especially those that lines critical engine components (Figure 11C). Sound energy can induce vibrations and resonance in components, which can subsequently generate heat due to energy dissipation. By incorporating sound‐absorbing lattices close to engine components, it is possible to dampen these vibrations and minimize the undesirable effects of excess heat and operational instability. Similarly, aircraft cabins can also benefit from these metamaterials to improve passenger comfort and safety. Under normal use, they serve as effective materials for noise control within the cabin. Also, they can be used in seat cushions and interior linings to absorb impact energy during turbulence or hard landings.

## 11 Conclusion

The emergence of additive manufacturing has bestowed the capability to fabricate intricate structures, thereby creating exciting opportunities for the development of sound absorbers. In this comprehensive review, we have highlighted the potential of lattice structures as cutting‐edge and promising materials for sound absorption. This is primarily attributed to their exceptional design flexibility, enabling customizable pore morphology and interconnectivity, which greatly enhances their acoustic performance. Thus far, the sound absorption properties of most of the types of lattice structures have been studied. Notably, lattice structures have demonstrated favorable performance compared to commercial absorbers, as they operate on the same absorption principles while offering the additional advantage of customizable properties through structural design.

We have reviewed the various sound absorption mechanisms observed in lattice structures. These include the Helmholtz resonance, cavity resonance, effective property, and slow‐sound effect models. Additionally, we have examined the FEA methods used for modelling sound absorption curves and studying dissipation in lattice structures. Following these, we have thus brought forward a novel classification for lattice structures in terms of their acoustically active geometry and mechanisms. In terms of acoustical mechanisms, at the unit cell level, lattice structures can be classified into three primary types, those with i) small pores and large cavities, ii) uniform and continuous cavity, and iii) uniform pores and cavities. For lattices with small pores and large cavities, sound dissipation takes place via Helmholtz resonance, akin to Helmholtz resonators. In turn, cavity or structural resonance takes place for lattices of only uniform and continuous cavity. Owing to the resonance mechanisms, sound absorption curves of these structures are based on characteristic resonance peaks. For lattices with uniform pores and cavities, sound absorption curves are in turn of a more smooth and gradual nature. Beyond the unit cell, heterogeneous combinations of cells, such as i) heterogeneous assembly in parallel, ii) functionally graded, and iii) combination of different mechanisms, are also possible. In these cases, the sound absorption curve is usually a superposition of its constituents which thereby translates to improved absorption bandwidth. Despite their complexity, lattice structures can typically be accurately modelled in terms of sound absorption curves using appropriate acoustical impedance models that focus on a few critical acoustical geometries. In terms of acoustics, lattice structures are characterized and defined by four features, namely their i) morphology, ii) relative density, cell size, and iv) number of cells along the sound propagation direction. These factors have a direct impact on the acoustical geometries that sound waves propagate through and the overall depth of sound wave dissipation in the medium. Notwithstanding, the relation between these features and absorption behavior is usually complex.

The current research on sound‐absorbing lattices represents only a fraction of all the possibilities. Despite this, it paves the way for further exploration. Several new concepts and advancements include leveraging structural design and concept, materials selection, and additional functionalities. For instance, the design principles utilized for mechanical design can be adapted for sound‐absorbing lattice structures. These principles encompass unit cell design inspired by various concepts, as well as macro‐level designs involving heterogeneous, fractal, hierarchical, and graded structures. As demonstrated in this review, sound absorption is independent on the material used to fabricate the lattice. Researchers can also explore the selection of materials that best cater to specific multifunctional applications. For instance, lattices can be fabricated using advanced materials, such as carbon‐derivatives, aerogels, or composites, to potentially achieve enhanced sound dissipation. The use of stimuli‐responsive materials also enables smart and active absorbers. By incorporating novel materials, alongside their meta‐mechanical properties, a broader range of applications, such as electromagnetics or thermal dynamics, can also be introduced to lattice structures.

## Conflict of Interest

The authors declare no conflict of interest.
